# Three Types of Collateral Arterial Supply to the Spleen After Spleen-Preserving Distal Pancreatectomies with Splenic Vessels Resection—How to Use This Knowledge for Organ(s) Preservation in Locally Advanced and Borderline Resectable Pancreatic Head Cancers Surgery—Hemodynamic, Surgical and Oncological Outcomes of 134 Spleen-Preserving Pancreatectomies

**DOI:** 10.3390/cancers18101675

**Published:** 2026-05-21

**Authors:** Viacheslav Egorov, Soslan Dzigasov, Alexey Kolygin, Mikhail Vyborniy, Grigoriy Bolshakov, Roman Petrov, Pavel Kim, Anna Demchenkova, Alexander Sorokin

**Affiliations:** 1 Ilyinskaya Hospital, 143421 Moscow, Russia; s.dzigasov@ihospital.ru (S.D.); koligin@gmail.com (A.K.); m.vyborniy@ihospital.ru (M.V.); gregbol19912020@gmail.com (G.B.); petrov-r-v@yandex.ru (R.P.); drpoul@yandex.ru (P.K.); demchenkovaanna89@gmail.com (A.D.); 2 Burnasyan State Research Center of the Federal Medical Biological Agency, 119435 Moscow, Russia; 3 National Medical Research Centre “Treatment and Rehabilitation Centre”, Federal State Autonomous Institution, Ministry of Health of the RF, 125367 Moscow, Russia; 4 Department of Mathematical Methods in Economics, Plekhanov Russian University of Economics, 117997 Moscow, Russia; alsorokin@statmethods.ru

**Keywords:** locally advanced pancreatic cancer, arterial resection, Warsaw procedure, arterial collaterals, blood flow adaptation, spleen preservation, splenic artery resection

## Abstract

**Background**: Spleen-preserving (SP) distal pancreatectomy (DP) with splenic vessels resection (SVR) (Warsaw procedure, WP) is an option for the treatment of tumors with low malignant potential. The reverse blood flow through the short gastric arteries (SGA) explains the preservation of the spleen after SVR, but leaves the source of the blood supply to the SGAs hidden. The types of blood supply to the spleen after WP and their incidence have not been previously described, nor has the significance of these types for locally advanced pancreatic head cancer (LAPHC) surgery been determined. **Aim**: To determine the main types of spleen blood supply after WP, and to assess the feasibility and safety of splenic artery (SA) rotation for the organ-preserving surgery of LAPHC. **Methods**: Retrospective analyses of demographic and perioperative data, including CT scans, overall (OS) and progression-free (PFS) survival after 71 SP DP SVR and 41 SP SVR pancreaticoduodenectomies (PD) and total pancreatectomies (TP) for LAPHC (2007–2025). **Results**: In 134 SP procedures, SA was resected in 115 cases (71DP, 9 TP, 3 central, and 32 PD). Indications for surgery were MCN (41), IPMN (14), CSA (3), NEN (25), SPPN (8), PHDAC (40), sarcoma (1), autoimmune (1), and calculous chronic pancreatitis (1). There were no deaths or ischemia-related splenectomies. Morbidity—31% (n23); Dindo–Clavien (D-C) > 3b-2.8%; POPF-grade B-n7 (10.6%); splenic infarctions on CT after SVR-n18 (23%), one symptomatic. CT revealed three types of arterial blood supply to the spleen after SPDP SVR: left gastric artery (LGA) type (n50, 70, 5%), gastro-epyploic arcade (GEA) type (n9, 12, 5%), and an intermediate type (n12, 17%). Spleen- and pancreas tail-preserving SVR pancreatectomies for LAPHC (n41) were accompanied by rotation of the SA to substitute resected SMA (n19) and CHA (n15) for 26 Whipples and 8TPs. There were no ischemic complications. D-C > 3–19.5%. Median OS and PFS for PDAC were 35 and 21 months for 29.5 months median follow-up. **Conclusions**: Despite the preservation of blood flow through all potential sources of splenic blood supply following resection of the splenic artery, the main collaterals supplying the spleen after WP are LGA branches (~90%). This knowledge, with strict adherence to the developed criteria, allows for the safe preservation of the spleen, pancreatic tail, and stomach during pancreatectomies with SA resection, including its rotation for the substitution of the SMA and CHA in LAPHC.

## Simple Summary

Short gastric arteries (SGAs) are considered the basis of spleen preservation in the Sutherland–Warshaw procedure, but the sources of blood supply to the SGAs are not described. This work showed that after this surgery (1) three types of collateral blood supply to the spleen are observed: left gastric (LGA), gastroepiploic arcade (GEA) and mixed. (2) LGA plus mixed types’ incidence is ~90%, and spleen can be preserved following the excision of up to 10 cm of the splenic artery (SA). The excision of long SA fragments and its rotation to replace excised superior mesenteric or common hepatic arteries can be safely performed with spleen and distal pancreas preservation in surgery for locally advanced pancreatic head cancer (LAPHC). Revascularization of the SA after resection of the celiac trunk and LGA can preserve not only the spleen and distal pancreas, but also the stomach. Using the blood flow through the collateral network “LGA-branches–SGAs–LGEA–SA” can allow for radical LAPHC operations to also be organ-preserving.

## 1. Introduction

Almost half a century has passed since spleen-preserving (SP) distal pancreatectomy (DP) with splenic vessels resection (SVR) was described by David Sutherland in 1980 [1], and popularized by Andrew Warshaw [2]. Preservation of the spleen during DP reduces the risks of postoperative complications, postoperative pancreatic fistula, overwhelming postsplenectomy sepsis, increased susceptibility to severe infections, thromboembolic events and certain solid and hematologic malignancies; improves the immunologic, hematologic and hemodynamic consequences of these interventions; reduces hospital stay; and decreases readmission rates [3,4,5,6,7,8,9].

The Sutherland–Warshaw method has proven to be safe and effective for the surgical treatment of benign pancreatic diseases and pancreatic tumors with low malignant potential [3,5,10], and, subject to certain conditions, in the treatment of neuroendocrine tumors and pancreatic body ductal adenocarcinoma [10,11,12]. The main area of application of this operation is circumstances that allow for spleen preservation for oncological reasons, but do not allow for preservation of the splenic vessels due to their involvement in the tumor or inflammatory process.

If, during SPDP with splenic vessels preservation (SVP), as described by Mallet-Guy [13] and later by Kimura [14], the source of arterial blood supply to the spleen remains the main vessel—the splenic artery (SA), in the case of SPDP SVR—the blood supply to the spleen can be explained by reverse blood flow through the short gastric arteries (SGAs) [15]. This explanation is beyond doubt, but questions about which collateral(s) deliver(s) blood to the SGAs after excision of the SA remain unanswered.

Despite the large number of publications and meta-analyses comparing the surgical consequences of SPDPs with and without SVR [4,16,17,18,19], the answer to the above question has not been given, although knowledge of the types of collaterals supplying the system of the SGAs and spleen after SA resection could (1) make SPDP SVR safer; (2) prevent splenic ischemia during subsequent surgical interventions; (3) and provide grounds for the safe performance of spleen- and distal pancreas-preserving right-sided and total pancreatectomies.

The purpose of this work was to study (1) the types of collateral blood supply to the SGAs and spleen after SPDP SVR; (2) the surgical consequences of spleen- and pancreas-preserving pancreatectomies with SVR through assessment of their short- and long-term morbidity; and (3) the feasibility and outcomes of using collateral pathways to the system of SGAs for radical and organ-preserving surgery for locally advanced (LA) pancreatic head cancers (PHCs).

## 2. Methods

### 2.1. Study Design and Objective

Ethical approval was obtained from the Ethics Committee of Ilyinskaya Hospital, Moscow, Russia (No. 12-02-SA/2021; approved 14 May 2021); the study was performed according to the principles of the Declaration of Helsinki [20] and Strengthening the Reporting of Cohort and case Series Studies in Surgery criteria, and is reported in line with the PROCESS and STROCSS Guideline for case series [21,22] (Files S1–S4).

### 2.2. Patient Population

Between December 2007 and December 2025, 134 consecutive patients underwent spleen-preserving pancreatectomy with or without splenic vessels resection. Of these, 115 underwent procedures that included splenic vessels resection and 19 underwent spleen-preserving pancreatectomy without splenic vessels resection. Procedures were analyzed in two clinically distinct cohorts:

1. **Distal pancreatectomy cohort**—spleen-preserving distal pancreatectomy with splenic vessels resection.
2. **Extended resection cohort**—pancreaticoduodenectomy or total pancreatectomy for LA and BR pancreatic head cancers with preservation of the spleen or/and distal pancreas and stomach using collateral perfusion pathways.

For this study, splenic vessels resection required resection of at least the splenic artery. Procedures involving isolated splenic vein resection without splenic artery resection, e.g., WATSA [23], were excluded.

### 2.3. Perioperative Evaluation, Patient’s Selection and Treatment Strategy

All patients were reviewed in a multidisciplinary conference. The operative intent in each case was organ preservation whenever feasible without compromising oncologic principles or vascular safety.

Patients with pancreatic ductal adenocarcinoma (PDAC) underwent staging with contrast-enhanced CT or MRI, tumor markers (CA 19-9 and CEA), and PET-CT to exclude distant disease. All patients with PDAC received neoadjuvant chemotherapy with FOLFIRINOX or a modified FOLFIRINOX regimen. Borderline resectable tumors generally received 6 cycles and more advanced tumors 12 cycles, with additional treatment individualized according to biologic response, biliary decompression needs, or interval reassessment. Restaging was performed approximately 3 weeks after chemotherapy completion, and surgery proceeded in the absence of progression and with a favorable treatment response. Mandatory conditions for selecting patients for arterial resections were a test of time from 8 to 10 weeks, a significant decrease in or normalization of blood CA 19-9, physical status ECOG 0, achieved with or without prehabilitation, good mental status, and the presence of a rehabilitation team capable of solving the short- and long-term problems of the patient.

Postoperative chemotherapy for PDAC was either gemcitabine, gemcitabine + abraxane, or mFOLFIRINOX. One FOLFIRINOX (mFOLFIRINOX) cycle was defined as 2 weeks, one gemcitabine-based cycle as 3 weeks per standard dosing. Radiation therapy was not used either before or after surgery.

Demographic and perioperative data of the patients undergoing SP pancreatectomies were retrospectively explored from medical records, follow-up charts, and CT-diagnostic reports. Tumor size delineated in mm was measured on CT before surgery and at pathohistological examination after surgery. Postoperative complications were graded according to Clavien–Dindo as minor (<Grade 3) or major (>Grade 3) [24]. Postoperative pancreatic fistula (POPF) was defined according to the International Study Group on Pancreatic Fistula classification [25], and post-pancreatectomy hemorrhage (PPH) was determined by guidelines given by the International Study Group of Pancreatic Surgery [26]. Splenic infarctions or abscesses were considered as surgery-related ischemia. Complications, readmissions, and mortality were collated up to 90 days postoperatively.

Survival data were collected based on the last CT or MRI results, last visit to the hospital, or follow-up phone calls. Patients who were alive at the time of data collection were included in survival analysis after no less than 24 months follow-up. Overall (OS) and progression-free (PFS) survival are presented for pancreatic ductal adenocarcinoma and were measured from the date of tissue diagnosis until death or unless otherwise specified [27,28].

A negative pancreatic resection margin in all cases was confirmed intraoperatively using a frozen-section biopsy (Intraoperative R0). Resectability was determined by CT data before treatment according to the National Comprehensive Cancer Network guidelines for pancreatic adenocarcinoma. Version 2.2023 [29].

### 2.4. Operative Decision Model: Criteria for Adequate Collateral Perfusion

The operative decision to preserve the spleen after splenic vessels resection was based on a structured assessment of whether collateral circulation could provide sufficient arterial inflow and venous drainage. After temporary clamping of the splenic artery at its origin and at the planned distal transection level, perfusion was reassessed over time. Spleen preservation proceeded only when predefined indicators of viability were present.

**Adequate splenic perfusion required the following intraoperative findings:**

**Figure 1:**
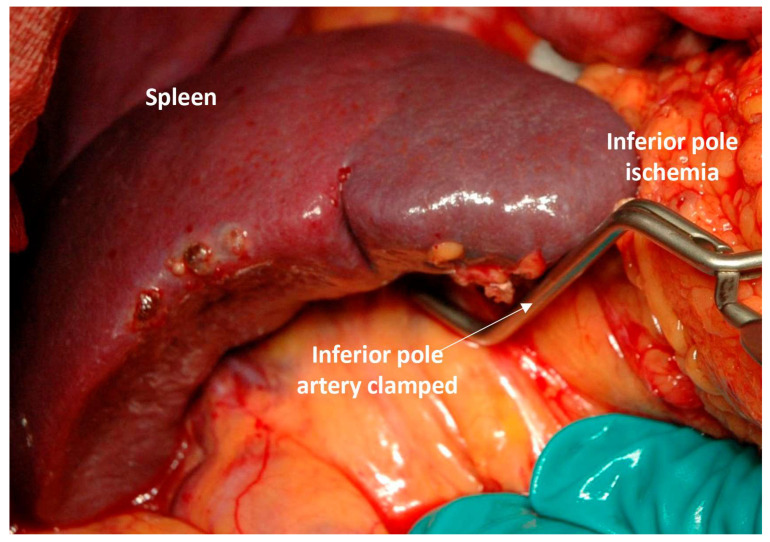
A test to evaluate the arterial blood supply to the spleen after clamping the splenic artery. This is used in cases when clamping of the splenic vein leads to darkening of the spleen, which makes it difficult to assess the adequacy of its arterial blood supply by color change. In these cases, temporary (15–20 min) clamping of one of the branches of the terminal splenic arteries leads to a change in the color of its blood-supplied area to a darker color compared to the remaining surface of the spleen (as shown in the photo). This means that the arterial blood supply to the spleen is preserved and it can be spared.

1. **Distal arterial pulsation**—a palpable or visible pulse in the remaining distal segment of the splenic artery 15–30 min after clamping, indicating effective collateral arterial inflow. This technique was not used in minimally invasive pancreatic resections.
2. **Preserved splenic appearance**—maintenance of acceptable splenic color and consistency. Significant darkening, cyanosis, or marked swelling/congestion was considered evidence of inadequate perfusion and a contraindication to preservation.
3. **Intraparenchymal arterial flow on ultrasound**—detection of arterial signals within the splenic parenchyma by intraoperative ultrasonography was considered evidence of viability [30].
4. **Retrograde back-bleeding from the distal splenic artery stump**—after release of the clamp from the remaining distal arterial segment, visible retrograde flow was interpreted as confirmation of collateral filling of the splenic arterial tree (Video S1).
5. **Selective polar branch occlusion test (when perfusion was uncertain)**—if global splenic darkening after vessels clamping made assessment difficult, a soft vascular clamp was placed on a terminal splenic branch (typically a lower-pole vessels). If the dependent splenic sector became darker within 15–20 min while the remaining spleen stayed perfused, collateral arterial supply to the unclamped parenchyma was considered adequate (Figure 1).
6. **When the pancreatic tail was also being preserved**, adequacy of remnant pancreatic perfusion required fulfillment of the first four splenic criteria plus arterial bleeding from the freshly transected pancreatic stump, indicating preserved blood supply to the distal pancreas (Video S2).
7. A mandatory requirement for **performing PD with resection of the splenic artery with its rotation** was (a) a hard pancreas, a (b) wide (at least 5 mm) pancreatic tail duct, and removal of the pancreatic body.

If these criteria were not met, or if findings remained equivocal, spleen and distal pancreas preservation was not pursued.

### 2.5. Surgical Technique for Spleen-Preserving Distal Pancreatectomies with and Without Splenic Vessels Resection

Pancreatic resections were done via an open or minimally invasive (laparoscopic or robotic) approach. The splenic artery (SA) was divided in all cases before reaching the origin of the left gastroepiploic artery if the latter originated from the SA. Pancreatic transection was performed using either a harmonic scalpel or a linear stapler. In open procedures, the pancreatic stump was routinely covered with the round ligament of the liver. Operative drains were placed selectively using passive or active systems. For benign and low-grade malignancies (neuroendocrine (NEN) or solid-pseuodopapillary tumors (SPPT)), pancreatic transection margins were minimized while maintaining a negative frozen-section margin. The most critical criterion for inclusion in the study was the preservation of blood flow through all potential sources of splenic blood supply following resection of the splenic artery, namely, the right and left gastroepiploic arteries, the gastroepiploic arcade, the short gastric arteries, the left gastric artery, and its branches.

**Surgical Technique for Pancreatic Head Cancers**

Surgeries for locally advanced (LA) and borderline resectable (BR) cancers were undertaken only after exclusion of distant metastases and careful assessment of the possibilities of (multi)vascular reconstruction. Diagnostic laparoscopy was performed during the same procedure, followed by midline or bilateral subcostal laparotomy, if no metastases or positive peritoneal washing cytology were found. Laparoscopy with peritoneal washings was undertaken in cases of equivocal imaging or unexplainably elevated CA 19-9, large primary tumors, or large regional lymph nodes. Intraoperative ultrasound, and frozen-section biopsy were used in cases of doubtful structures in the liver or peritoneum. The necessary technical conditions for arterial and venous revascularization were sufficient diameters of the distal targets, which were determined with high accuracy on CT. In all LA cancers we used the “arterial resection and reconstruction before mobilization of the pancreas” (ARRBMP) method, i.e., the arterial hepatic or intestinal revascularization was performed before the part of the pancreas containing the tumor and the involved artery was mobilized. Resections included pancreatoduodenectomy and total pancreatectomy with associated lymphadenectomies. En bloc resection of vascular structures was performed with revascularization determined by anatomical and organ-preserving necessity. The reconstruction of portal (PV), superior mesenteric vein (SMV), or PV/SMV confluence included end-to-end primary anastomoses or the interposition of synthetic graft. In the case of resection of the splenic vein, if it was of sufficient length, it was sutured into the left renal vein; if the stump was short, reconstruction of the splenic vein was not performed. En bloc arterial resections included the hepatic artery, celiac axis, superior mesenteric artery, or multiple arterial resections. Arterial revascularizations included end-to-end primary anastomoses or interposition grafts via the great saphenous vein. In case of total pancreatectomy, the SA was divided before reaching the origin of the left gastroepiploic artery, if the latter originated from the SA. If preservation of the tail of the pancreas was expected, the splenic artery was divided before reaching the origin of a. pancreatica magna.

If it was necessary to remove the left hepatic artery (LHA) without reconstruction due to its small diameter in addition to the main surgery, then the left liver lobe was thoroughly examined using IO Doppler US after the temporary clamping of the LHA. Transection of the lobar artery was performed only after IOUS definite confirmation of intramural arterial blood flow within the entire liver [31]. In the absence of intraparenchymal arterial blood flow in the segments of the left lobe, the ischemic segments were removed.

All operations were performed under 3.5–4.5 magnification.

**Computed Tomography and CT Angiography Protocol**

Multiphasic contrast-enhanced computed tomography (CT) and CT angiography (CTA) were central to both preoperative planning and postoperative evaluation of collateral circulation. Imaging was used not only to stage disease, but also to define the vascular anatomy before and after surgery to assess the collateral arterial pathways to the organs preserved after splenic vessels sacrifice.

### 2.6. Preoperative Imaging Assessment

CT and CTA were obtained within 3 weeks before surgery, or closer to the operative date when clinically indicated. Preoperative studies were systematically reviewed for the following:

1. **Tumor characterization**—lesion type, size, location, morphology, and radiographic features suggestive of benign, low-grade, or invasive malignant disease.
2. **Tumor-vessels relationships**—degree and length of contact, abutment, narrowing, encasement, or occlusion involving the splenic artery, splenic vein, celiac axis, common hepatic artery, superior mesenteric vessels, and adjacent venous structures. This analysis informed resectability and anticipated need for vascular resection [32].
3. **Baseline arterial anatomy**—classification of hepatic and upper abdominal arterial variants according to Michels [33], with specific attention to vessels that could contribute to collateral perfusion after splenic vessels resection.
4. **Collateral reserve mapping**—caliber (diameters) and continuity of the left gastric artery (LGA), right gastroepiploic artery (RGEA), left gastroepiploic artery (LGEA), short gastric vessels, and other perigastric pathways potentially available for splenic reperfusion.

### 2.7. Postoperative Imaging Assessment

Follow-up CT or CTA was performed between postoperative days 3 and 30, or earlier when prompted by clinical concern. Postoperative imaging was analyzed for the following:

1. **Procedure-related complications**—fluid collections, hemorrhage, abscess, thrombosis, pancreatitis, anastomotic or stump-related complications, and other adverse findings.
2. **Collateral perfusion pathways**—demonstration of the dominant arterial routes supplying the spleen, or the spleen and preserved pancreatic remnant, after splenic vessels resection. Particular attention was paid to flow through the gastric and gastroepiploic arcades.
3. **Splenic ischemia**—presence, distribution, and volumetric extent of splenic infarction or abscess formation.
4. **Venous adaptation or congestion**—development of gastric, perigastric, or epigastric varices as radiographic markers of altered venous drainage.
5. **Vascular remodeling**—interval change in the diameter of major collateral vessels, including the LGA, RGEA, and LGEA. Measurements were obtained 10 mm distal to the vessel origin or immediately proximal to the first major branch point to standardize comparison between preoperative and postoperative studies.

### 2.8. Imaging Rationale

This imaging strategy was designed to test the study hypotheses by linking **preexisting vascular anatomy**, **postoperative collateral recruitment**, and **clinical outcomes**. In practical terms, CT and CTA were used to determine not simply whether the spleen survived, but *how* it remained perfused and which vascular configurations were most reliable for safe spleen-preserving surgery.

### 2.9. Quantitative Hemodynamic Analysis

To estimate adaptive changes in collateral circulation, preoperative and postoperative vessel diameters were compared. Based on Poiseuille’s principle, relative changes in vessel radius were used as a surrogate for changes in potential flow capacity, recognizing the limitations of applying simplified fluid models to biologic vessels. Small-vessel measurements were enhanced using semiautomated vascular segmentation techniques.

Poiseuile’s law (Q = πΔPr^4^/8ηL), for the laminar, one-dimensional, incompressible flow of a Newtonian fluid within a rigid circular tube (a rough approximation for an artery) shows that volumetric flow rate (blood flow intensity, Q, in mL/min) is directly proportional to the pressure difference (ΔP) and the fourth power of the deformed luminal radius, and inversely proportional to viscosity (η) and vessel length [34,35]. With the viscosity of blood, and blood pressure and the length kept constant before and after surgery, a comparison of arterial radii after and before surgery makes it possible to estimate blood flow intensity changes through the measured arterial segment with acceptable accuracy (Q_after_/Q_before_). The accuracy of small-diameter blood vessels (from 1 mm) measurements was ensured by applying the segmentation of vascular structures and integral metrics method. The level set method was used for blood vessel segmentation, which was performed semiautomatically in two steps: semi-automatic segmentation with the fast marching method and final segmentation with the geodesic active contours method [36].

### 2.10. Assessment of Outcomes

Outcomes were selected to directly test whether collateral pattern predicts operative safety and long-term feasibility.

#### 2.10.1. Primary Outcomes

- Successful spleen and distal pancreas preservation without unplanned splenectomy or/and pancreatectomy.
- Splenic ischemia (infarction or abscess).
- Major postoperative morbidity (Clavien–Dindo grade III or higher) [24].

#### 2.10.2. Secondary Outcomes

- Postoperative pancreatic fistula (ISGPS definition) [25].
- Postpancreatectomy hemorrhage [26].
- Readmission within 90 days.
- Mortality within 90 days.
- Development of gastric/perigastric varices.
- Endocrine function (postoperative diabetes mellitus assessed ≥3–4 months after surgery using ADA criteria) [37].
- Overall survival and progression-free survival for PDAC patients.

Signs of spleen infarction included (1) low-density parenchyma on non-contrast CT scans, (2) no contrast enhancement in arterial and/or venous phases (3) and lesion of more than 1% of the total spleen parenchyma (3D imaging data). Transabdominal Doppler ultrasound was performed for three postoperative days unless necessary later. All patients received proton pump inhibitors for three months. Follow-up consisted of physical examination, laboratory studies, and MRI/CT imaging at 3-month intervals for the first two years, at 6-month intervals for years 3 through 5, and then at yearly intervals.

The clinical and CT data of the patients presented in [30] were re-evaluated and included in this publication.

**Postoperative Diabetes Mellitus Assessment**

Examinations to detect diabetes were carried out no earlier than 3–4 months after surgery, after the formation of eating habits using American Diabetes Association criteria for diabetes mellitus. The diagnosis required one of the following laboratory findings: hemoglobin A1c (HbA1c) ≥ 6.5% (≥48 mmol/L), fasting plasma glucose ≥ 126 mg/dL (≥7.0 mmol/L), two-hour plasma glucose (2 h PG) ≥ 200 mg/dL (≥11.1 mmol/L) during glucose tolerance test, and random plasma glucose ≥ 200 mg/dL (≥11.1 mmol/L) in the presence of classic symptoms of hyperglycemia or hyperglycemic crisis [37].

### 2.11. Pathology

For pancreatic malignancy resection margins, including transection and circumferential margins, were categorized according to the Royal College of Pathologists’ definition and classified as R0 (no residual tumor, distance margin to tumor ≥ 1 mm), R1 (residual tumor, distance margin to tumor < 1 mm), and R2 (residual tumor, macroscopically positive margin) [38].

### 2.12. Statistical Analysis

Analyses were performed using IBM SPSS Statistics version 27. Continuous variables were summarized using mean ± standard deviation or median (interquartile range), depending on distribution. Categorical variables were reported as counts and percentages. Group comparisons used Student’s *t* test or Mann–Whitney *U* test for continuous variables and χ^2^ or Fisher’s exact test for categorical variables. Associations between vascular patterns and outcomes were explored using correlation and subgroup analyses. Survival outcomes were estimated by the Kaplan–Meier method and compared with the log-rank test. Statistical significance was defined as *p* < 0.05.

## 3. Results

Indications for surgery were tumors with low malignant potential and benign diseases in 93 (69.4%) cases, and PDAC and MPNST in the remaining 41 (30.6%) cases. Indications for surgery and types of operations are presented in Table 1.

In accordance with the objectives of the study and the scope of application of the interventions, all operations were divided into two groups: SP distal pancreatectomies and other SP pancreatectomies. There was no postoperative mortality. The follow-up period ranged from 9 months to 19 years.

### 3.1. Spleen-Preserving Distal Pancreatectomies (SPDP)

SPDP were performed only for benign lesions and tumors with low malignant potential. In all cases the patients’ preoperative physical status was defined as ASA I-II. Table 2 presents indications, perioperative and demographic data; Table 3 presents morbidity.

Young women predominated among those operated on. Fifty-five operations were performed openly, 15 laparoscopically, and 13 were robotically assisted. With a median follow-up of 88 [58; 116] months, four (5%) patients were lost to follow-up more than 8 years after surgery. Seven patients died 4, 5, 6, 7, 7, 7, and 12 years after surgery, two from unknown causes, the rest from recurrent brain tumor due to VHL, colorectal cancer, ovarian cancer, breast cancer, and myocardial infarction.

In all nine patients with a bdIPMN, tumors were multiple and localized in all parts of the pancreas. The operation was performed for tumors localized in the body–tail of the gland and showed 2–3 worrisome features [39]. During the follow-up of these patients after surgery, there were no signs of the development of cancer in the head of the gland or other reasons for re-surgery of the pancreas.

During the follow-up after SPDP, sixteen patients underwent abdominal operations without surgical complications: for endometrioid cysts (3), right hemicolectomy, oophorectomy (2), bilateral adrenalectomy (VHL), panhysterectomy, sigmoidectomy, cholecystectomy (3), and kidney resection (VHL). There were no re-operations on the pancreas.

Five patients with NEN had hereditary tumor syndromes: MEN 1 (n3) and VHL (n2). In three of them (2NEN and 1VHL), the tumors were multiple and were located in all parts of the gland. During the observation period, unremoved non-functioning NENs in the head of the pancreas did not require re-surgery due to the small tumor size.

In 71 SPDP SVRs (SDC. Results, Table 1), the splenic vein was preserved in eight cases (11%). In the remaining 63 cases, 17.5% of patients developed gastric/epigastric varices of varying degrees as a manifestation of left-sided portal hypertension. This complication resulted in recurrent gastric bleeding only once (1.6%), which required splenectomy after unsuccessful attempts to embolize the branches of the left gastric artery.

The most common complication was pancreatic fistula, occurring at a rate of 47.5% (including POPF Grade A). In 16 (19%) cases, it corresponded to Grade B, due to prolonged drainage (n15) or its re-installation (n1). The incidence of POPF did not differ between the groups with and without SVR.

Late complications occurred in two patients after SPDP SVP, against the background of splenic vein thrombosis; bleeding from esophageal varices was reported once, and once, progressive splenomegaly occurred. In both cases, the splenic artery embolization 6 and 12 months after surgery resolved the problem.

Splenic infarctions accounted for 9–35% of splenic volume and occurred in 18 patients after SPDP SVR (25%). All of them were asymptomatic both in the short- and long-term postoperative period.

**Figure 2:**
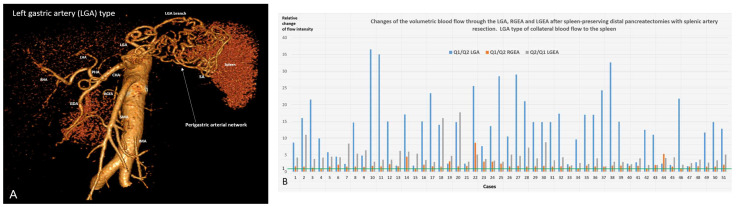
LGA type of collateral blood supply to the spleen after SPDP SVR. (**A**) CT angiographic (CTA) demonstration of the left gastric artery (LGA) type of blood supply to the spleen after SPDP SVR for mucinous cystadenoma in a 35-year-old woman. The short gastric arteries, and through them, the spleen, receive blood from the branches of the LGA. In this case, the diameter of the LGA after surgery increased 1.9 times, which increased the volumetric blood flow (VBF, Q) through the artery by 13 times. The diameter of the left gastroepiploic artery (LGEA) increased by 2 times, and through this, it increased by 16 times. The diameter of the right gastroepiploic artery (RGEA) did not change significantly. The trunk of the splenic artery (SA) is fully excised; gastroepiploic arcade was not closed before and after surgery. (**B**) The chart of calculated relative change in blood flow intensity (volumetric flow rate in mL/min, Q) through the LGA, RGEA and LGEA after SP DP. As shown, the blood flow intensity through the LGA can increase by 2.5–36 times and LGEA by 4–36 times (with regard to the baseline, i.e., the 1.0 value), which underlines the adaptive ability of the LGA branches and their hemodynamic contribution to this type of collateral blood supply to the spleen. The CTA shows case #18.

**Figure 3:**
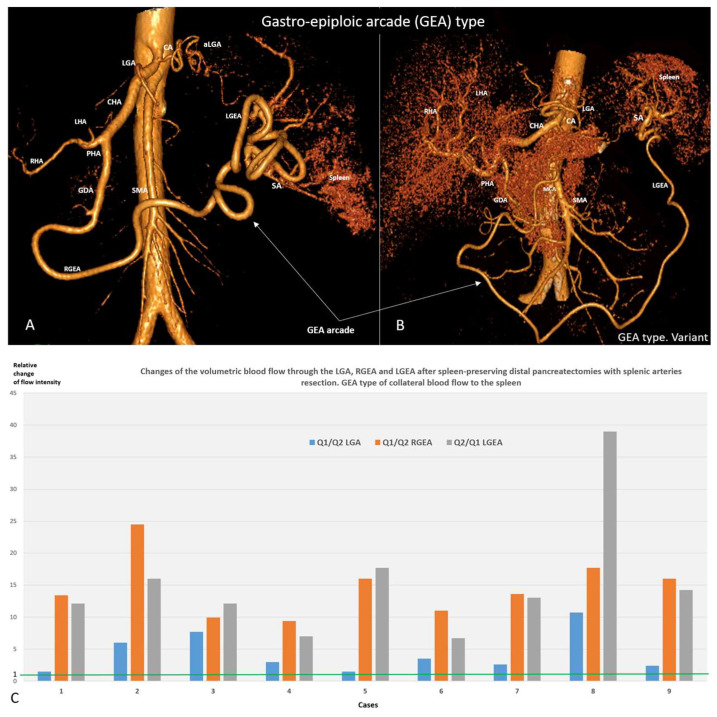
GEA type of collateral blood supply to the spleen after SPDP SVR. (**A**,**B**) CTA demonstration of the gastro-epiploic arcade (GEA) type of blood supply to the spleen after SPDP SVR for mucinous cystadenomas in 25- (**A**) and 41- (**B**) year-old women. The short gastric arteries, and through them, the spleen, receive blood from the gastroepiploic arcade. The gastroepiploic arcade, which was open before surgery, is now closed. In case A, the trunk of the SA is fully excised, and the diameter of the LGA after surgery increased insignificantly and is half the diameter of the RGEA. In both cases, RGEA and LGEA increased their diameters by two times, increasing the VBF through each artery by 16 times after surgery. In case B, six cm of the SA trunk was excised and RGEA originated from the middle colic artery, which is a described but rare variant. (**C**) The chart shows the calculated relative change in blood flow intensity (volumetric flow rate in mL/min, Q) through the LGA, RGEA and LGEA after SP DP. As shown, the blood flow intensity through the GEA can increase by 8–24 times and LGEA by 7–38 times (with regard to the baseline, i.e., the 1.0 value), which underlines the adaptive ability of the GE arcade and its hemodynamic contribution to this type of collateral blood supply to the spleen. The CTA shows cases #2 and #5.

**Figure 4:**
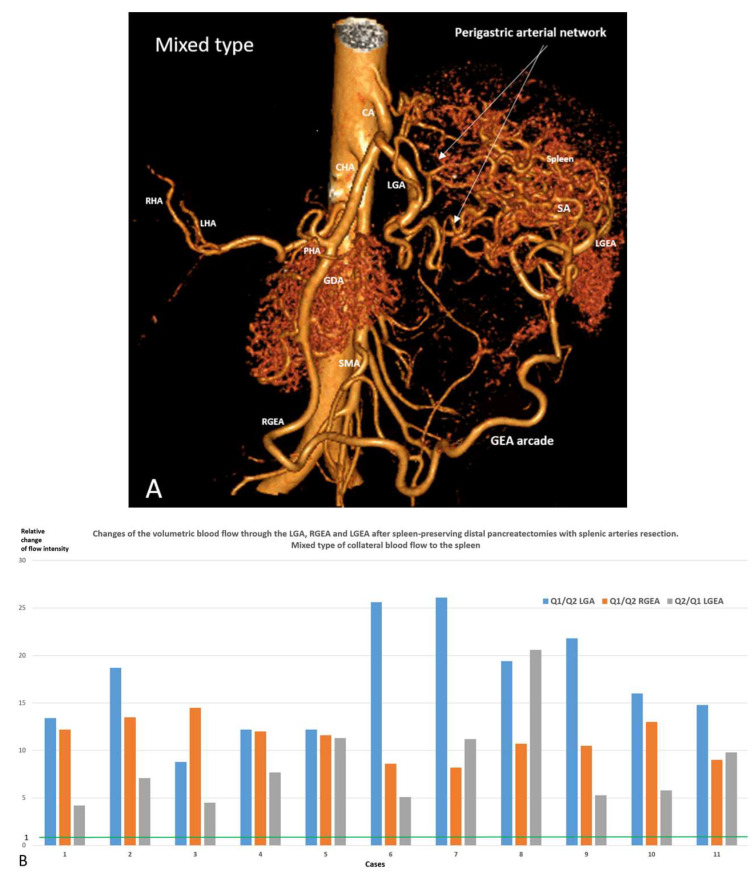
Mixed type of collateral blood supply to the spleen after SPDP SVR. (**A**) CTA demonstration of the mixed type of blood supply to the spleen after SPDP SVR for mucinous cystadenoma in a 52-year-old woman. The short gastric arteries, and through them, the spleen, receive blood from the gastro-epiploic arcade and from the branches of the LGA. In this case, the diameter of the LGA, RGEA and LGEA after surgery increased significantly (1.5–1.95 times), which means the VBF increased through the arteries from 9.4 to 14.5 times. The trunk of the SA was fully excised, and the gastroepiploic arcade, which was not closed before surgery, closed. (**B**) The chart shows the calculated relative change in blood flow intensity (volumetric flow rate in mL/min, Q) through the LGA, RGEA and LGEA after SP DP. As shown, the blood flow intensity through the LGA can increase by 8–26 times, RGEA by 7.7–14 times, and LGEA by 4–21 times (with regard to the baseline, i.e., the 1.0 value), which underlines the adaptive ability of the LGA and GE arcade and the united hemodynamic contribution of this type of collateral blood supply to the spleen. The CTA shows case #11. Abbreviations for the figure legends: VBF—volumetric blood flow; CA—celiac artery; SMA—superior mesenteric; SA—splenic; LGA—left gastric; RGEA—right gastro-epiploic; LGEA—left gastro-epiploic; RHA—right hepatic; LHA—left hepatic; GDA—gastroduodenal arteries.

Postoperative CT revealed three types of arterial blood supply to the spleen after SPDP SVR: left gastric artery (LGA) type when LGA was the main collateral (n51.72%) (Figure 2A,B), gastro-epyploic arcade (GEA) type (n9, 12.7%) (Figure 3A–C), and an intermediate (mixed) type (n11, 15.3%) (Figure 4A,B) when both collateral pathways are determined. The variant of blood supply to the spleen from the SMA system (from a. colica media to the left gastro-epiploic artery) after resection of the splenic vessels (Figure 3B), was found in one patient with GEA type (Table S1).

In the LGA type, the key feature was an open gastro-epiploic (GE) arcade and a significant increase in diameters of the LGA and LGEA, demonstrating a 2.5–36 times increase in the blood flow intensity through the LGA and LGEA (Figure 2A,B). Minimal changes in volumetric blood flow through the LGA (<4.5 times) were noted when the excision of an SA segment was less than 6 cm in length (14 of 16 cases) (Table S1).

The key feature of the GEA type was a closed GE arcade with a significant increase in the diameters of the RGEA and LGEA, with a slight expansion of the LGA and an undeveloped arterial network running from this to the spleen. The blood flow intensity through the RGEA and LGEA increased by 7–38 times (Figure 3A–C).

In the mixed type, the GE arcade was closed, and there was a simultaneous significant increase in the diameters of LGA, RGEA and LGEA, through which the blood flow intensity increased 4–26 times (Figure 4A,B).

The degree of increase in the diameter of the artery (and, consequently, the degree of increase in volumetric blood flow through it) for any type of collateral blood flow was greater with a smaller initial diameter of the vessel (Table S1).

### 3.2. Spleen-Preserving Pancreatectomies with Preservation of the Splenic Vessels Other than Distal Resections Were Performed in Seven Cases

Total pancreatectomy (TP) was performed in six cases for mdIPMN (with one instance being duodenum-preserving), and once for a neuroendocrine tumor on the head of the pancreas against the background of multiple bdIPMNs of the body and tail. Once, a center-sparing resection of the pancreas was performed for multiple NENs in VHL syndrome. In the latter case, POPF grade B was noted due to the long-standing drain.

### 3.3. Spleen-Preserving Pancreatectomies with Resection of the Splenic Vessels Other than Distal Resections

**Figure 5:**
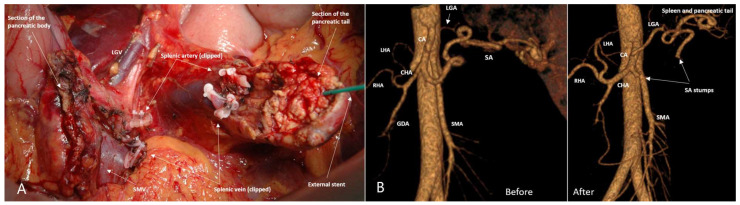
Central pancreatectomy with splenic vessels resection for G2 neuroendocrine tumor in a 41-year-old lady. (**A**) An operating field after completion of the resection stage; (**B**) CTA after surgery, showing the sources of blood supply to the pancreatic tail and spleen from the branches of the LGA, the diameter of which has increased by two times, which means the VBF through the LGA increased by 16 times. Gastroepiploic arcade was open before and after surgery.

Central resections were performed in three young women for SPPT (1) and NEN (2) with an uneventful short- and long-term postoperative period (Figure 5A,B).

### 3.4. Spleen-Preserving Pancreatectomies with Resection of the Splenic Artery (Vessels) for Borderline Resectable and Locally Advanced Pancreatic Head Cancers

**Figure 6:**
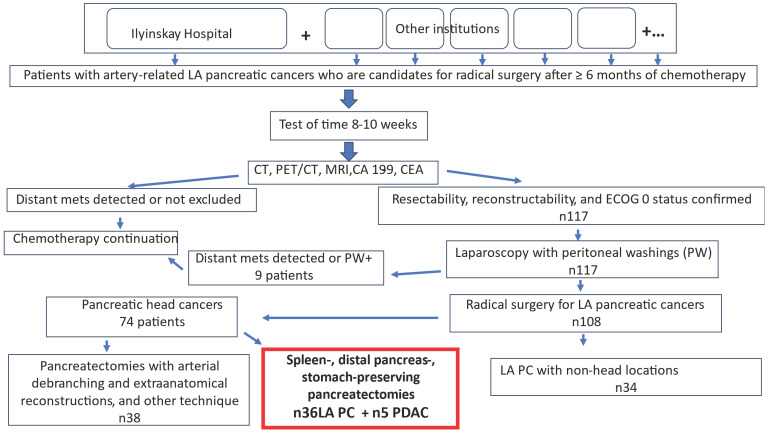
Flow chart of selection of patients with locally advanced pancreatic cancer for radical surgery. The study cohort is shown in the red rectangle.

From December 2013 to December 2025, 117 patients with LA PC were operated on; in nine cases, distant metastases or positive peritoneal washings were identified, which excluded these patients from candidacy for radical surgery. One hundred and eight patients with LA PC underwent radical surgery, 74 of whom had LA PHC. In 36 cases of LA PHC with splenic vessel resection, spleen-, distal pancreas-, and stomach-preserving procedures were used (Figure 6), and this series was supplemented by five patients with spleen- and distal pancreas-preserving TP and PD with the splenic vessel resection performed for BR PDAC, as shown in Table 4 and Table S2. SP TP and PD 41 SVR present the perioperative data of this cohort. In the remaining cases of LA PHC, a different surgical technique was employed. (Figure 6). Chronic oxaliplatin-induced peripheral neuropathy (grade 3 in two patients) was the main manifestation of chemotherapy toxicity, but the test of time in all cases allowed patients to approach surgery with minimal symptoms.

**Figure 7:**
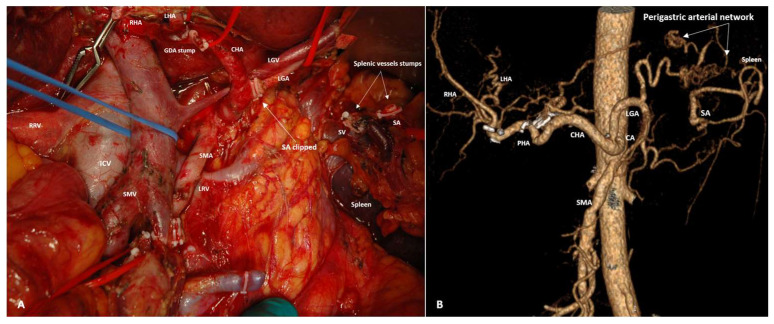
Total pylorus- and spleen-preserving pancreatectomy with splenic vessels resection for pancreatic head ductal adenocarcinoma (PHDAC) against the background of MD IPMN in a 51-year-old woman. (**A**) An operating field after completion of the resection stage; (**B**) CTA after surgery, showing the sources of blood supply to the spleen from the branches of the LGA, the diameter of which increased by 1.8 times, increasing the VBF through the LGA by 10.5 times.

**Figure 8:**
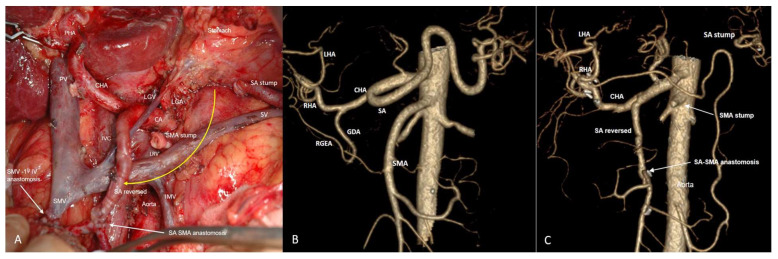
Total pylorus- and spleen-preserving pancreatectomy with SMA and SMV resection, resection and rotation of the splenic artery (SA) for the substitution of the SMA and SA-SMA anastomosis for LA PHDAC in a 73-year-old man. (**A**) An operating field after completion of the resection stage. Yellow arrow shows the clockwise rotation of the SA. (**B**,**C**) CTA before (**B**) and after (**C**) surgery, showing the postoperative sources of blood supply to the spleen from the branches of the LGA, the diameter of which increased by 1.6 times, increasing the VBF through the LGA by 7.4 times.

SP TP SVR for pancreatic head cancer was performed because of concomitant mdIPMN and the tumor was inseparable from the SA (n2) (Figure 7A,B), and fragile pancreatic remnant (n6) occurred in case of SA rotation (Figure 8A,B).

**Figure 9:**
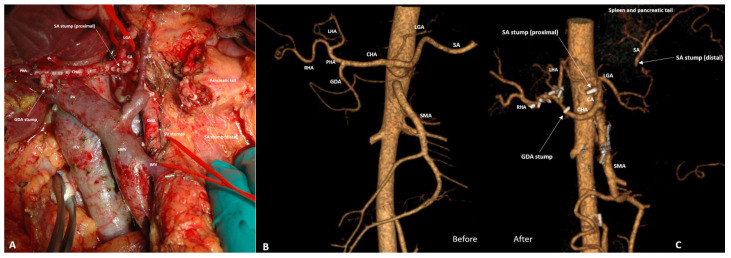
Pylorus-preserving pancreaticoduodenectomy with splenic vessels resection for BR PHDAC in a 54-year-old lady. (**A**) An operating field after completion of the resection stage. CTA before (**B**) and after (**C**) surgery, showing the sources of blood supply to the pancreatic tail and spleen from the branches of the LGA after splenic vessels resection. The diameter of LGA increased by two times, increasing the VBF through the LGA by 16 times.

Three SP PD SVRs were performed due to the inability to separate SA from the tumor (Figure 9A–C).

**Figure 10:**
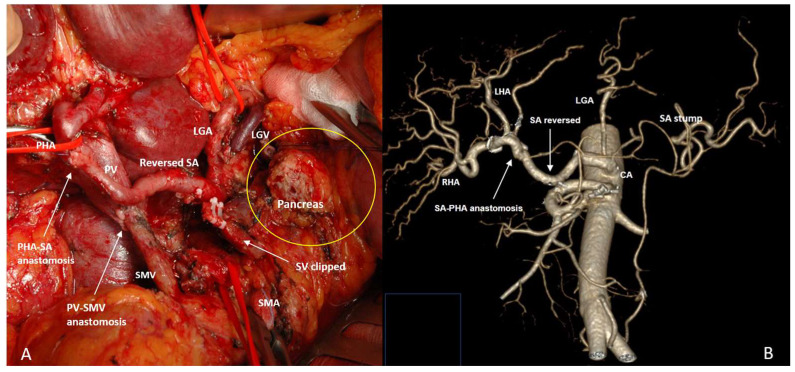
Pylorus-preserving pancreaticoduodenectomy with CHA, SMV and SV resection, spleen- and distal pancreas-preserving resection and rotation of the splenic artery (SA) for SA-PHA anastomosis for PHDAC with CHA, SMV and SV involvement in a 66-year-old man. (**A**) An operating field after completion of the resection stage. The SA is rotated counterclockwise and anastomosed with proper hepatic artery (PHA); the pancreas is shown by the yellow circle. (**B**) CTA after surgery, showing the sources of blood supply to the spleen and the pancreatic tail from the branches of the LGA, the diameter of which increased by 1.5 times, increasing of VBF through the LGA by 5 times.

**Figure 11:**
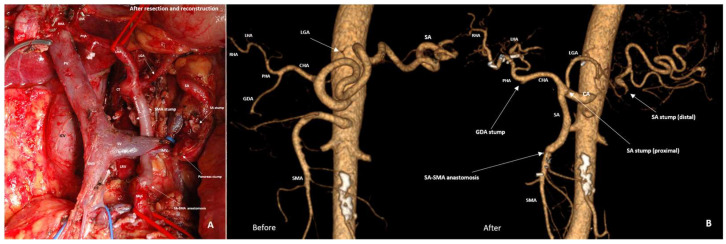
Pancreaticoduodenectomy with SMA and SMV resection, spleen- and distal pancreas-preserving resection and rotation of the splenic artery (SA) for SA-SMA anastomosis for PHDAC with SMA and SMV involvement in a 65-year-old woman. (**A**) An operating field after completion of the resection stage. The SA is rotated clockwise and anastomosed with the SMA. Resection of the posterior wall of the SMV. The SV is pulled down by the blue vessel loop to expose the SMA stump. (**B**) CTA shows the sources of blood supply to the spleen and pancreatic tail from the branches of the LGA after surgery, the diameter of which increased by 1.8 times (**B**), increasing the VBF through the LGA by 10.5 times.

Twenty-eight PDs were performed with splenic artery rotation for LA PHC involving the CHA (n15) and/or SMA (n19) (Figure 10 and Figure 11).

**Figure 12:**
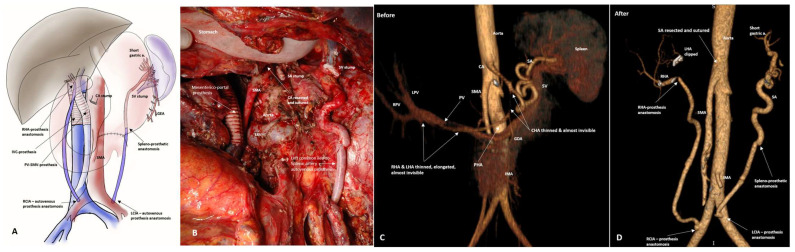
Removal of the recurrent gigantic MPNST in a 33-year-old lady with stomach- and spleen-preserving total pancreatectomy, right nephr- and adrenalectomy, and resection of the CA, SA, LGA, common, proper, left and right hepatic arteries, portal, splenic and inferior cava veins. Cava-caval and SMV-PV synthetic prostheses. Right common iliac to right hepatic artery and left common iliac to splenic artery autovenous prostheses. Spleen and the whole stomach were preserved after resection of the CA with the left and right gastric and RGEA due to revascularization of the SA remnant, which supplies the SGA-LGEA network. (**A**) A scheme of surgery after reconstruction of blood vessels and gastrointestinal tract: (**B**) An operating field after completion of the resection stage; (**C**,**D**) CTA before and after surgery, showing the new sources of the spleen and stomach blood supply.

**Figure 13:**
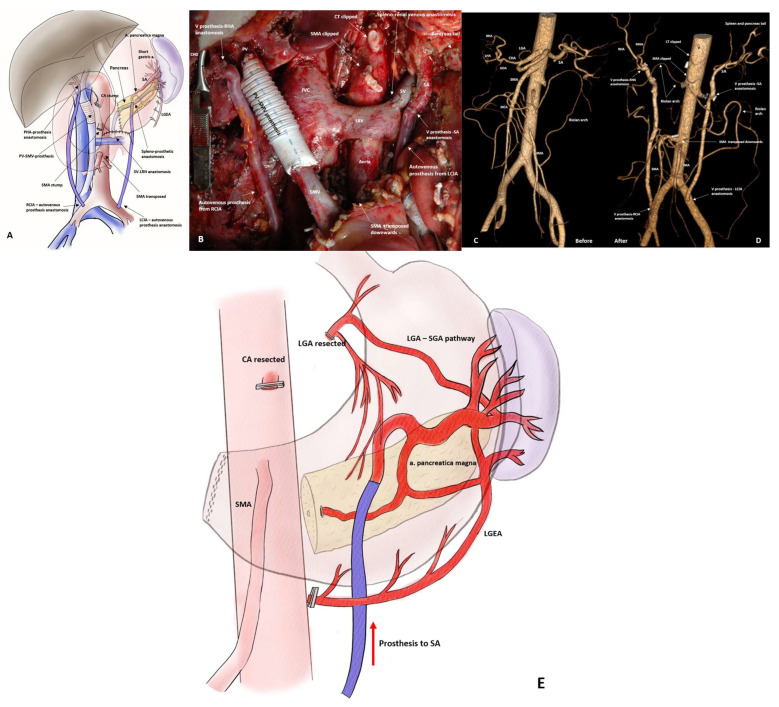
LA PDAC in a 42-year-old lady after 12 courses of FOLFIRINOX. Pancreaticoduodenectomy, SII-III liver segmentectomy with resection of CA, SMA, LGA, SA, right and middle colic, and both hepatic arteries, portal, superior and inferior mesenteric, splenic, left gastric, and left portal veins. Transposition of the SMA into the lower aorta. Arterial right common iliac-to-right hepatic and left common iliac-to-splenic autovenous prostheses. Splenorenal venous anastomosis. PV-SMV alloprosthesis. Spleen, distal pancreas and the whole stomach were preserved after resection of the CA with the left and right gastric arteries and RGEA due to revascularization of the SA remnant, which supplies the SGA-LGEA network. (**A**) A scheme of surgery after reconstruction of blood vessels and gastrointestinal tract. (**B**) An operating field after completion of the resection stage. (**C**,**D**) CTA before and after surgery, showing the new sources of the spleen and stomach blood supply, (**E**) Schematic representation of the arterial collateral network “Splenic artery–Short gastrics–LGEA–LGA”. During resection of the celiac trunk and/or LGA, the use of this collateral network makes it possible to preserve not only the spleen and tail of the pancreas, but also the entire stomach. The diagram is also correct for Figure 11, in which the pancreas is completely removed but the stomach is preserved due to the mechanism described above.

In two cases, resection of the celiac trunk and all its branches, including the LGA and not only the spleen and part of the pancreatic tail, but also the stomach, were preserved due to revascularization of the splenic artery with an autovenous graft from the left common iliac artery (Figure 12 and Figure 13A–D). Table 4 presents the demographic and perioperative data of patients with locally advanced pancreatic tumors (n41).

**Figure 14:**
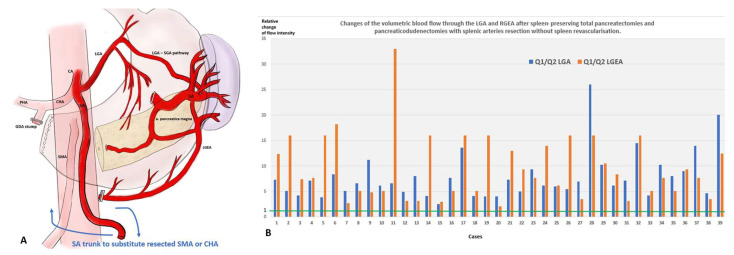
(**A**) Schematic representation of the arterial collateral network “LGA–Short gastrics–LGEA–SA–a. pancreatica magna”. During resection of the SA, the use of this collateral network made it possible to preserve not only the spleen but also the tail of the pancreas. The straightened trunk of the splenic artery is long enough for rotation clockwise or counterclockwise to replace the SMA or CHA. The blood supply to the spleen and tail of the pancreas in these cases occurs due to collateral from the LGA; (**B**) LGA type of collateral blood supply to the spleen and distal pancreas after total pancreatectomies and pancreaticoduodenectomies with splenic vessels resection. The chart shows calculated relative change in blood flow intensity (volumetric flow rate in mL/min, Q) through the LGA and LGEA after surgery. As shown, the blood flow intensity through the LGA can increase by 3.5–26 times and LGEA by 3–33 times (with regard to the baseline, i.e., the 1.0 value), which underlines the adaptive ability of the LGA branches and their hemodynamic contribution to collateral blood supply to the spleen after the above-mentioned surgery.

In all cases of BR and LA tumors of the head of the pancreas, after TP and DP SVR, the blood supply to the spleen and distal pancreas was preserved due to significant dilatation of the LGA and its branches, which increased the blood flow intensity through the LGA and LGEA from 3 to 33 times (Figure 14).

The physical status of all patients before surgery was defined as ECOG 0 and ASA II. The reason for resection of the splenic vessels without rotation of the splenic artery when performing PD (n3), central (n3) or TP (n2) was suspicion of tumor involvement in the SA on CT, and the inability to separate this from the tumor at surgery, which in most cases was combined with the spread of the PHDAC to the pancreatic body (n5). The R0 resection level was achieved in 92% of patients, the R1 resection points were found at the SMA margins in all cases.

After SP DP and TP SVR, clinically significant complications developed in 19.5% (Table 5).

Two patients underwent relaparotomy: one for perforation of a duodenoenteroanastomotic ulcer (POD 17) and one for splenic rupture due to continued thrombosis of the resected splenic vein (POD 12). In one case, narrowing of the hepaticojejunostomy required dilatation and transhepatic biliary drainage, and in one case, diagnostic angiography for hypocoagulative bleeding was performed.

Ischemic events were limited to five splenic infarctions of less than 10% volume, none of which were clinically significant. Grade B pancreatic fistula, caused by prolonged drainage, occurred in one patient after PD with resection of the SMA (7.4%).

Diabetes mellitus was detected preoperatively in four of 41 patients with LA PHC patients, and developed postoperatively in seven of 31 patients after SPPD SVR (22.6%). In three of these seven cases, the tail of the pancreas was also resected. In four cases, compensation for diabetes was achieved with glucose-lowering tablets, and in three with insulin therapy. There was no significant deterioration in the course of diabetes mellitus identified before surgery.

**Figure 15:**
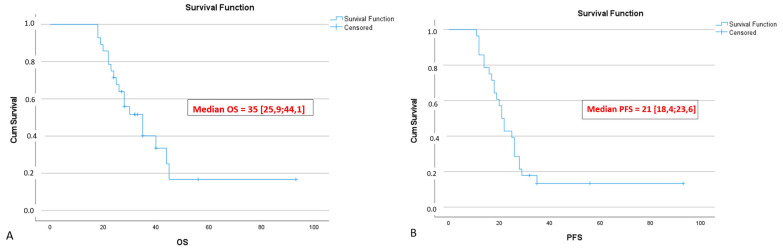
Overall (**A**) and progression-free (**B**) survival after spleen-preserving total pancreatectomies and pancreaticoduodenectomies with splenic vessels resection for BR and LA PDAC.

With a median follow-up of 29.5 [25.5; 37.0] months for PHDAC patients, median OS was 35 [26; 44] months, and median PFS was 21 [18.4; 23.6] months (Figure 15A,B).

## 4. Discussion

**Spleen-Preserving Distal Pancreatic Resection (SPDP SVR):** After SPDP and other SVP procedures, the sources of arterial blood supply to the spleen do not require explanation; however, for SPDP SVR this issue is little studied. General considerations regarding the participation of the short gastric arteries [15] in this process require documentation of the sources of blood delivery to these vessels, since previous work explored, to a greater extent, the potential significance of the existing highways, mainly the LGEA and the gastroepiploic arcade [30,33,40,41]. The present study provides the first analysis of the types of collateral arterial blood supply to the spleen after SPDP SVR and their incidence. Despite the preservation of blood flow through all potential sources of splenic blood supply following resection of the splenic artery, after SPDP SVR, blood to the SGA is most often (72%) delivered through the branches of the LGA, and much less often through the gastro-epyploic arcade (12.7%), and in 15.3% of cases, both of these pathways are involved. That is, in 87% of SPDP SVR cases, compensation of the arterial inflow to the spleen occurs due to the LGA and its branches.

From a hemodynamic point of view, after blockade of the main artery, to ensure collateral blood flow, it is more advantageous to increase the diameter of one (single) vessel than to include several vessels with a diameter equal to the original one. The hemodynamic benefit of the sole collateral instead of two or more is well documented [34,35], and this rule works well for the LGA and GEA types of blood supply to the spleen. The mixed type is not so easy to explain, but it is possible if we take into account the variants of weak communication between the blood vessels supplying the upper and lower poles of the spleen [33]. Anatomical studies [33] show that the GE arcade is closed in 90% of cases. In SPDP SVR it is used as a key collateral in 27% of cases (Figure 3 and Figure 4). That is, in no less than 63% of cases, the GE arcade does not become the leading collateral to the spleen, despite the fact that it is closed and reaches the distal part of the splenic artery, i.e., is anatomically capable of delivering blood to the SGA system and spleen. This once again demonstrates the principle “a single collateral is better than two or more” [34,35]. Modern CT using the segmentation method [31] makes it possible to accurately determine the diameters of even small arteries and calculate changes in volumetric blood flow when the cross-section of the vessel changes. It is the change in volumetric blood flow, and not its absolute value, that characterizes the ability of collateral vessels to adapt supply in cases of main line blockage.

The collateral system of all types demonstrated a high adaptive ability to reorganize blood flow: the volumetric blood flow through these arteries was able to increase by 2.5–38 times. Minimal changes in volumetric blood flow through the LGA and its branches in the LGA type were noted when excised segment of SA was less than 6 cm in length. In this case, it is likely that pre-existing collaterals between the proximal and distal segments of the SA are used, particularly those between the LGA and the posterior gastric artery (SDC. Results, Table 1).

It is interesting that in all three cases of central pancreatectomy with resection of the splenic artery (vessels), despite the preservation of all sources of collateral blood flow, the main collateral was the LGA and its branches (Figure 5), although there was not a significant increase in the diameter of the LGA. This fact once again emphasizes the dominant role of the LGA-type collateral pathway and confirms the principle of a hemodynamic benefit of a sole collateral instead of two or more.

Knowledge of the types of collateral arterial blood supply to the spleen is important: firstly, to understand the need to preserve all potential collateral routes of blood supply in SPDP SVR, because it is impossible to determine the type of collateral anatomy before surgery; secondly, to plan subsequent interventions in the pancreas, stomach or colon, because in these cases, the new blood supply to the spleen may be damaged. Given the young age of patients undergoing these surgeries and the long survival period [15,16,17,18,19], the likelihood of new surgery is high and this risk is not theoretical. In the present study, after 16 abdominal surgeries performed years after SPDP SVR, there were no ischemic complications of the spleen. These surgeries included two hemicolectomies, but there were no operations on the pancreas or stomach.

Thirdly, the knowledge that after SPD SVR, in almost 90% of cases, the main artery supplying the spleen is the left gastric artery and that the SGA network works well in both directions provides a sufficient basis for us to introduce operations with resection of the splenic vessels and preservation of not only the spleen, but also the distal pancreas and stomach for locally advanced pancreatic cancers.

**Pancreaticodudenctomies and Total Pancreatectomies with Splenic Vessels (Artery) Resection for Borderline Resectable and Locally Advanced Pancreatic Tumors**

In patients with LA-PDAC with a definite response to chemotherapy, surgical resection significantly improves survival compared with continuation of chemotherapy [42], as shown in large surgical series [27,42,43,44,45,46,47,48]. Surgical techniques that provide wide clearance from the tumor borders and a high rate of R0-resections, and which feature simplicity and reliability of vascular resections and reconstructions, along with the highest possible organ preservation, acquire special meaning in the conditions of complex surgery of LA PDAC.

The SPDP SVR experience shows that the spleen can be preserved by excision of a long (up to 100 mm) segment of the splenic artery (SDC. Results, Table 1). It turns out that such a fragment of the artery can be safely removed, preserving not only the spleen, but also the tail of the pancreas, supplied by the a. pancreatica magna through the SGA–LGEA system (Figure 5, Figure 6, Figure 8, Figure 9 and Figure 10). We successfully used this knowledge to preserve the spleen and tail of the pancreas in LA PHDAC and to replace the removed segments of the SMA and CHA by rotating the splenic artery around the site of its origin (Figure 5, Figure 6, Figure 7, Figure 8, Figure 9 and Figure 10) before mobilizing the pancreatic tumor, as well as to preserve the stomach by restoring blood flow through the LGEA–SGA system during CA resections. The feasibility of spleen-preserving total pancreatectomy with splenic vessels resection (SPTP SVR) was demonstrated by Sutherland [49] and later by Yang [50]. Yang’s et al. (2018) [50] series of 38 patients with borderline resectable pancreatic cancer was the first example of the systematic use of SPTP SVR in pancreatic cancer surgery. In this work, two splenectomies for malperfusion (5%) confirmed our data on the various pathways of collateral blood supply to the spleen after resection of SA, and the absence of clinically significant ischemic complications (31.4% of asymptomatic splenic infarctions), as well as significant dilation of the LGA after surgery, which confirmed the reliability of the main collateral pathway to the SGA and spleen through the LGA and its branches in the majority of cases. With a 90-day mortality rate of 5.4%, despite a high R0 resection rate (97.4%) at a median follow-up of 12 months, the median overall (OS) and relapse-free survival (RFS) after SPTP SVR were higher than after conventional TPs (16 and 12 months), but not significantly. At the same time, higher values for the horizontal part of the RFS plot compared to OS indicate a relatively high level of non-cancer mortality in the comparison groups. Overall, the work did not reveal significant differences in the rate of complications and survival after SPTP SVR and standard TP.

In a series of 18 patients with borderline resectable and unresectable (UICC 7) pancreatic cancer (2014), Mizuno, Desaki et al. [51,52] first demonstrated the possibility of safe use of PD with splenic artery resection. Ten years later, another series of 40 PDs from Taiwan with short-segment SA resection for resectable and BR PDAC [53] confirmed the safety of such interventions. Both of these series were free of mortality, without ischemic complications, with a major complication rate (D-C > 3) of less than 17%, and a rate of postoperative diabetes mellitus of 22% and 30%.

Our series of SP SVR for PHDAC included 5 PDs for BR PDAC (n5) and 36 surgeries for LAPH cancers: 34 procedures with resection and rotation of the SA to replace the SMA or CHA (8 SP TP and 26 SP PD) and two operations with revascularization of the splenic artery to preserve not only the spleen, but also the stomach and pancreatic tail. This series demonstrated the feasibility and safely of radical resections for LA PHC with resection of the CHA or/and SMA along with resection of extended SA fragments while strictly adhering to intraoperative procedure selection criteria. There were no deaths or ischemic complications after these extensive interventions; the only Grade B pancreatic fistula was due to prolonged drainage. Complications requiring relaparotomy were nonspecific to pancreatectomies with arterial resection. The most common complications were diarrhea (42%) and lymphorrhea (29%), although their frequency was not significantly different from that in a large series of operations for MR PDAC [41,42,43,44,45,46,47,48].

In PD and TP SVR, we considered the most important condition for sparing the spleen and pancreas tail to be the preservation of the LGA, the left inferior phrenic artery and their collaterals, since a significant increase in volumetric blood flow through the LGA in all cases in our series (Figure 14) definitely indicated that the main collaterals to the spleen and distal pancreas after SPTP and SPPD SVR are LGA and its branches.

Similar considerations are presented in References [51,52,53]. The Desaki and Mizuno series included two patients who previously underwent total gastrectomy, and one of whom also underwent splenectomy. The authors reasonably hypothesized that in the absence of an LGA and SA, the posterior epyploic artery (PEA) may be the source of blood supply to the tail of the pancreas, although the data presented did not exclude the involvement of the left inferior phrenic artery. We were unable to assess the value of PEA for preserving the pancreatic tail and spleen during LGA resection, because we could not count on PEA, the anatomy of which is unknown, as well as the possibility of its preservation during resection of the tail of the pancreas. In cases of resection of the LGA (with CA) without possibility of its reconstruction, the entire stomach, pancreatic tail and spleen were preserved by delivering blood to the network of the SGA and LGEA through revascularization of the splenic artery with an autologous vein from the left iliac artery (Figure 11 and Figure 12). Cases of SA revascularization during resection of the CA and LGA have shown that the stomach and tail of the pancreas can be spared by preserving the spleen and the SGA-LGEA network. This tactic has shown to be effective in observations other than our own [54,55], and may change approaches to celiac trunk resections of types 1C, 2A,B and 3B according to the Mayo classification [43].

Rotation (transposition) of the splenic artery. (We prefer the term rotation because it accurately describes the mechanism of movement of the distal end of a transected artery. Transfer of the origin of an artery is also a transposition, and this can create confusion.)

The advantages of SA rotation for SMA or (any) hepatic artery reconstruction are (1) the relative ease of execution even when using the method of resection and reconstruction of arteries before mobilization of the pancreas (as we do systematically), (2) that a segment of SA up to 9 cm long can be used, which is usually sufficient to replace a fragment of the resected artery, (3) the approximately equal diameter of the anastomosed arteries, and (4) that only a single (5) quick arterial anastomosis is required, with (6) an autogenous artery. The average time for anastomosis with the SA was 21.9 ± 1.5 min and there were no short- or long-term artery(ies)-related complications.

The splenic artery transposition for pancreatic cancer was described by surgeons of Bochum (Germany) in four TPs for the first time in 2010 [56] to replace resected CHA. Since then, it has been believed that SA rotation is possible and safe only after total pancreatectomy with splenectomy. PD with SA rotation to replace resected CHA was mentioned in an anecdotal report [57]. Our series of 8 SP TPs and 31 PDs with 34 SA rotations to replace resected CHA and SMA showed that if a number of prerequisites are met, SA rotation can be performed safely and systematically, not only preserving the spleen but also the pancreatic tail.

In LA PDAC surgery, we use the ARRBMP technique (see Methods), in which resection and reconstruction of the artery(s) are performed before mobilizing the pancreas. This approach preserves the oncological principles of “no touch” surgery and “en-block” tumor removal, reduces the number of “points of fixation” before the tumor mobilization, eliminates time pressure during arterial reconstruction, extends margins clearance and converts this unusual situation for surgeons into a familiar one: after arterial resection and reconstruction, all that remains is pancreatectomy with vein resection. This method requires vascular segments of sufficient length to bypass the portion of the pancreas containing the tumor. It turns out that by straightening the bends and spirals of the splenic artery, it is always possible to obtain a sufficient segment of artery (Figure 14A) to replace the affected CHA or SMA segments, while preserving not only the spleen, but also the tail of the pancreas.

Spleen preservation in PDAC makes special sense because meta-analyses and multicenter studies demonstrate that splenectomy is an independent risk factor for shorter overall survival in pancreatic cancer, and spleen-preserving operations are associated with significantly prolonged survival [58].

The average operative time of 560 ± 146 min in our series is average for large series of PE + AR for BR and LA PDAC [27,42,43,44,45,46,47,48]; it was 47 min less than that in the work of Desaki et al. [52] and 260 min more than in [53], procedures with greater complexity for the treatment of LA pancreatic cancer. The average blood loss of 358 ± 211 mL in our series was comparable to that with standard PD [59], significantly lower than with TP of any type [60], including large series of PE + AR [41,42,43,44,45,46,47,48], almost five times lower than in the work of Desaki [52], and two times lower than in [53]. Only nine patients required a transfusion of two to six units of red blood cells during surgery.

Surrogate markers of surgical quality (number of lymph nodes removed, rate of R0 resections) were on par with data from large series of surgery for LA PDAC [27,42,43,44,45,46,47,48]. Although the number of lymph nodes removed was approximately the same, the rate of R0 resections in our series was higher than in [52,53] (92% vs. 78% vs. 55%), probably due to wider clearance during arterial resection and longer neoadjuvant chemotherapy. The median overall and disease-free survival in our series was consistent with current data from large series of PE +AR in LA PDAC [27,42,43,44,45,46,47,48], and was higher than that of [52,53] (35&21 vs. 20.9&14.8 vs. not reached &14), with a median follow-up of 27 months vs. 18 months [53] vs. unspecified [52], although 88% of patients had LA PDAC in our group.

We believe that not only spleen preservation [58] and slight blood loss [59,60], but also organ preservation [61,62,63] contributed to the survival of our patients. In addition to the benefits of spleen preservation, an important advantage of organ preservation in pancreatectomies with arterial resections for LA PDAC is the chance for higher quality of life, which notably manifests in the ability of patients to better tolerate postoperative chemotherapy compared with patients after multivisceral resections.

After multivisceral and arterial resections for pancreatic cancer, more than 33% of patients overall fail to receive adjuvant chemotherapy, primarily due to impaired functional recovery from surgical complications [64]. The concept of preoperative therapy for LA PDAC suggests that “any form of adjuvant therapy is highly unlikely to oncologically salvage patients after such extended resections” [43], although the duration of chemotherapy in this category of patients is an additional factor in prolonging life [44]. Although a recent comparison of TP to PD found that despite TP patients having more comorbidities, longer surgeries (7.2 vs. 6 h), more vascular reconstructions (77% vs. 50.8%), and greater blood loss (1200 vs. 600 mL), adjuvant chemotherapy initiation rates were similar (66% vs. 76%, *p* = 0.156) and completion rates were comparable (69.4% vs. 74.1%, *p* = 0.578) in both groups [61], the ability to receive full adjuvant therapy may be lost when there is a combination of conditions, each of which can cause prolonged lymphorrhea and/or refractory diarrhea, such as a combination of total pancreatectomy (which can differ in complexity [60]), total or subtotal gastrectomy, extensive retroperitoneal dissection and extensive resection or skeletonization of the SMA [27,42,43,44,45,46,47,48,63,64,65,66]. According to large series of PE+AR for BR and LA PDAC, only 21–77% of patients received adjuvant chemotherapy, and less than half of them completed treatment [41,42,43,44,45,46,47,48,63,64]. In our study, 93% of patients received adjuvant chemotherapy, of which 82% completed it, 13% were in the process of continuing treatment, and only 5% did not complete it, receiving only four courses of gemcitabine.

Preservation of part of the pancreas, especially the distal part, makes a significant contribution to the prevention of diabetes or mitigation of its course after extensive resection of the pancreas for LA PDAC, which not only improves the quality of life, but reduces the risk of tumor progression [67]. The rate of new-onset diabetes mellitus of 22.6% in our series was comparable to that (14–24%) after standard PD [67,68,69,70] and differed little from the data of Desaki [52] and Kuo [53] series.

Modern diabetes technologies, including long- and short-acting insulin analogs, continuous glucose monitoring systems, multidisciplinary follow-up, insulin pumps and bihormonal artificial pancreas, have markedly improved metabolic safety after TP and reduced hypoglycemia compared to conventional diabetes care [71,72]. However, a systematic review (2019) of 1536 patients showed that the overall quality of life after TP is impaired even for resectable and BR cases, mainly due to the impact of diabetes-related morbidity and diarrhea [73,74]. In the context of surgery for LA PDAC, which is much more extensive than standard vein-related resections for BR PDAC, with the same safety and radicality of PD and TP, additional time and actions in the operating room aimed at preserving organs may outweigh the advantages of artificial pancreas systems in the long term (QoL, survival, economical ets.), and this subject still needs to be studied.

Limitations: The study was performed in a single institution, and it was retrospective with inherent biases. The study period is long, although approaches to surgery did not change throughout its entire duration. There are no exact data on the number of intraoperative splenectomies performed during distal pancreatectomies in case of failure to meet the conditions necessary, in our opinion, to preserve the spleen. In this study, there were no operations on the pancreas or stomach months or years after SPDP SVR, which does not allow us to assess the risk of splenic ischemia after these procedures.

## 5. Conclusions

The present study provides an analysis of the types of collateral arterial blood supply to the spleen after spleen-preserving distal pancreatectomies with splenic vessels resection and their incidence. Despite the preservation of blood flow through all potential sources of splenic blood supply following resection of the splenic artery, blood in the SGA and in the spleen after SPDP SVR most often (72%) enters through the branches of the LGA (LGA type) and much less often through the gastro-epyploic arcade (GEA type) (12.7%), and in 15.3% of cases both of these pathways are involved. That is, in 87% of SPDP SVR cases, compensation of arterial inflow to the spleen occurs due to the LGA and its branches. The collateral system, in all its types, demonstrated high adaptability: volumetric blood flow through collateral arteries was able to increase by 2.5–38 times.

Knowledge of the types of collateral arterial blood supply to the spleen is important for (1) understanding the need to preserve all potential collateral routes of blood supply in SPDP SVR, because it is impossible to determine the type of collateral anatomy before surgery; (2) planning subsequent interventions on the pancreas, stomach or colon, because in these cases, the new blood supply to the spleen may be damaged; (3) the introduction of procedures with resection of extended segments of the splenic vessels and preservation of not only the spleen, but also the distal pancreas and stomach in LA PHDAC; and (4) the safe rotation of the splenic artery to replace resected CHA and SMA in LA PHDAC with preservation of not only the spleen, but also the distal pancreas.

The intraoperative criteria used to determine organ viability ensured safe resection of the splenic artery in PD or TP while simultaneously preserving the spleen, distal pancreas and stomach in LA PHDAC involving CA/CHA/SMA.

Methods of using collateral blood flow in both directions through the SA–SGA–LGEA–LGA system are presented, which can be useful for organ-preserving procedures for LA PHDAC, which increases the likelihood of receiving postoperative chemotherapy, and thereby can prolong life.

Survival for LA PHDAC involving the SMA and CHA after organ-preserving pancreatectomies was at least noninferior to that after TP with arterial resections.

We hope that our experience will be helpful for pancreatic and general surgeons, and researchers focused on vascular adaptation.

## Figures and Tables

**Table 1:** Indications for surgery and types of spleen-preserving pancreatectomies.

Indications for SP Pancreatectomies/Diagnosis		Number
1	Mucinous cystic neoplasm (MCN)	41
2	Branch duct intraepithelial mucinous neoplasia (bdIPMN)	9
3	Main duct intraepithelial mucinous neoplasia (mdIPMN) *	10
4	Serous cystic adenoma (CSA)	3
5	Well-differentiated neuroendocrine neoplasms (NEN)	23
6	Solid-pseudopapillary neoplasm (SPPN)	8
7	Chronic calculous pancreatitis (CP)	1
8	Autoimmune pancreatitis (local form) (AIP)	1
9	Malignant peripheral nerve sheath tumor (MPNST)	1
10	BR and LA pancreatic head ductal adenocarcinoma (PHDAC)	40
	Total	137
**Types of Surgery**		**Number**
Spleen-preserving pancreatectomies with splenic vessels preservation (SPPE SVP)		19
1. Distal pancreatectomy (SPDP SVP)		12
2. Total pancreatectomy (TP SVP)		6
3. Center-preserving pancreatectomy with splenic vessels preservation		1
Spleen-preserving pancreatectomies with splenic vessels resection (SPPE SPR)		115
1. Distal pancreatectomy (SPDP SVR)		71
2. Central pancreatectomy with splenic vessels resection (CP SVR)		3
3. Total pancreatectomy with splenic vessels resection (TP SVR)		2
4. Total pancreatectomy with SMA (3) or CHA (3) resection and splenic artery rotation (TP SAR)		6
5. Total pancreatectomy with HA/CA/LGA resection and splenic artery revascularization.		1
6. Pancreaticoduodenectomy with splenic vessels resection (PD SVR)		3
7. Pancreaticoduodenectomy with SMA or CHA resection and splenic artery rotation (PD SAR)		28
8. Pancreaticoduodenectomy with SMA and CA/HA/LGA resection and splenic artery revascularization.		1
Total		134

* In three cases, PDAC was associated with mdIPMN, in which both diseases were considered.

**Table 2:** Spleen-preserving distal pancreatectomies. Patient demographics and perioperative data (n83).

Age (mean ± SD)	44.23 ± 13.9
Female gender, n (%)	61 (76.25%)
BMI (mean ± SD)	22.58 ± 3.2
Tumor size (mean ± SD) (mm)	61.88 ± 40.0
OP time (min) (mean ± SD)	208.54 ± 143.6
E blood Loss (mL) (mean ± SD)	108.05 ± 146
Mean hospital stay (days)	5.2 ± 0.75
Median F-up time (months)	88 [58; 116]

**Table 3:** Morbidity after spleen-preserving distal pancreatectomies.

Type of Surgery	#	POPF Grade B	D-C ≥ 3	Spleen Infarction (n,%, cr)	Any Gastric/Epigastric Varices	Late Morbidity *	Postop DM
SPDP SVR	71	14 (20%) of 33	0	18 (25%), 0 cr	11(17.5%) of 63	1	9 (13%)
SPDP SVP	12	2 (22%) of 7	1	0	1 (8%)	2	1 (8%)
Total	83	16 (19%)	1	18 (22%), 0 cr	12 (14.5%)	3	10 (12%)

SPDP SVR—spleen-preserving distal pancreatectomy with splenic vessels resection; SPDP SVP—spleen-preserving distal pancreatectomy with splenic vessels preservation; * complications which happened months or years after surgery (late bleedings followed by arterial embolization and/or splenectomy); cr—clinically relevant; POPF—postoperative pancreatic fistula; DM—diabetes mellitus; D-C—Dindo–Clavien surgical morbidity classification.

**Table 4:** Perioperative characteristics of the patients with locally advanced and borderline-resectable pancreatic head cancers who underwent spleen-, distal pancreas- and stomach-preserving pancreatectomies with splenic artery (vessels) resection (n41).

Age (Years)	57.8 ± 10.1 (33–73)
Gender (m/f)	18/23 (44%/56%)
PDAC/MPNST	40/1
Neoadjuvant chemotherapy (NACHT) (yes/no)	41/0 (100%/0)
Mean number of NACHT courses	11.6 ± 2.95
Adjuvant chemotherapy (ACHT) (yes/no)	38/3 (93%/7%)
Completed ACHT, n38	31 (82%)
Incomplete ACHT. In process/incomplete, n7	5/2 (13.5%/2.5%)
Mean number of ACHT courses	4.43 ± 2.56
CCI—2/3/4/5/(score)	5/19/14/3 (12%/46%/34%/8%)
OP time (min)	560 ± 146 (195–1260)
Estimated blood loss (mL)	358 ± 211 (100–1200)
Operative packed RBC transfusion, n (cases)	9 (22%)
No. of packed RBC units transfused, ≥2/>2, n	6/3
PV/SMV resection (yes/no)	38/3(93%/7%)
CA 19-9 before NACHT, U/mL, n40	308.7 ± 352
CA 19.9 before surgery, n40	49.5 ± 69.6
CA 19-9 decreasing ratio after/before NACHT, n40	6.3
Tumor size at pathology (mm), n40	34.6 ± 13.3 (0–81)
Tumor grade, G1,G2,G3, n40	3, 33, 4 (7.5%, 82.5, 10%)
Contact on CT > 180° with SA, HA/CA, SMA, CA&SMA	5, 15, 19, 2 (12%, 36.5%, 46.5%, 5%)
Arteries resected in addition to SA: HA/CA, SMA, CA&SMA	15/19/2
*LA tumors (T4)/BR tumors(T3)*	36/5
Arterial invasion at pathology (yes/no), n40	27/14 (66%/34%)
PV-SMV invasion at pathology yes/no), n40	34/4 (90.5%/9.5%)
Perineural invasion (yes/no) (n40)	34/6 (85%/15%)
R0/R1-resection	38/3 (92.7/7.3%)
Number of lymph nodes removed (n40)	37.6 ± 13 (21–75)
Lymph nodes involvement, pN0/pN1/pN2 (n40)	7/20/13 (17.5%/50%/32.5%)
Tumor regression score, 0/1/2/3, n40	2, 2, 14, 22 (5%, 5%, 34%, 56%)
Pancreatectomy type, n (%): PD/total pancreatectomy	32/9 (78%/22%)
Mean time for anastomosis with the SA (min)	21.9 ± 1.5 min
Recurrence type, n (% total, % all recurrence), n40	
Local	3 (7.5, 11.5)
Peritoneal	5 (12.5, 19)
Distant (liver, lungs, lymph nodes, bone)	18 (45, 69)
Multisite	8 (20, 30.8)
Vital status at last follow-up, n (%)	
Alive, with disease	8 (20)
Alive, no evidence of disease	14 (35)
Died	19 (47.5) *

*—one patient died of non-oncological reasons; CCI—Charlson comorbidity index; PV—portal vein; SMV—superior mesenteric vein; PDAC—pancreatic ductal adenocarcinoma; PD—pancreaticodudenectomy; SA—splenic artery; HA—any hepatic artery(ies); CA—celiac artery; SMA—superior mesenteric artery; NACHT—neoadjuvant chemotherapy; ACHT—adjuvant chemotherapy.

**Table 5:** Morbidity after spleen-, distal pancreas- and stomach-preserving pancreatectomies for locally advanced and borderline resectable pancreatic head cancers with splenic artery (vessels) resection (n41).

Complications, C-D < 3/III, IV	32/7/2 (88%/17%, 5%)
POPF, Grade B, n32	1 (3%)
Diarrhea (n)	17 (42%)
Length of stay (days)	16.4 ± 4.7 (9–29)
Lymphorrhea (n)	12 (29%)
cr DGE, Grade 2/3	8/2 (20%, 5%)
Postpancreatectomy hemorrhage (n)	1 (2.4%)
Any cr ischemic abdominal complications	0
Reoperation (n)	2 (7.5%)
Readmission (n)	7 (17%)
Postoperative diabetes mellitus, n31	7 (22.6%)
Mortality, 90 days	0

C-D—classification of surgical morbidity by Clavien–Dindo; POPF—postoperative pancreatic fistula; cr—clinically relevant; DGE—delayed gastric emptying.

## Data Availability

The data supporting the reported results can be found in archived datasets of the Ilyinskaya Hospital and the Bakhrushin Brothers Moscow City Hospital. Data are available upon reasonable request to the corresponding author.

## Supplementary Materials

The following supporting information can be downloaded at: https://www.mdpi.com/article/10.3390/cancers18101675/s1, File S1: Report of the Ethics Committee; File S2: Consent; File S3: PROCESS 2023 guidelines checklist; File S4: STROCSS 2024 guidelines checklist; File S5: Statistics. TP and PD for LA PHDAC; Table S1: SPD SVR 71; Table S2: SP TP and PD 41 SVR; Video S1: Collateral blood flow test 1; Video S2 Collateral blood flow test 2.

## Presentation

Preliminary results of the study were presented at the 51st Annual Meeting of the European Pancreatic Club in Bergen, Norway, 26–29 June 2019, the 41st Annual Meeting of the German Pancreas Club, Lübeck, Germany, 10–12 February 2022, the 15th IHPBA World Congress, New York, NY, USA, 30 March–2 April 2022, the REDISCOVER International Consensus Meeting, Pisa, Italy, 17–18 September 2023, the 57th Annual Meeting of the European Pancreatic Club, Dusseldorf, Germany, 2–5 July, the Digestive Disease Week 2026, Chicago, IL, USA, 3–5 May.

## Abbreviations

The following abbreviations are used in this manuscript:

AChT	adjuvant chemotherapy
BR	borderline resectable
CA19-9	carbohydrate antigen 19-9
CA	celiac artery
CHA	common hepatic artery
LA	locally advanced
LGA	left gastric artery
LGEA	left gastro-epiploic artery
MPNST	malignant peripheral nerve sheath tumor
NAChT	neoadjuvant chemotherapy
OS	overall survival
PD	pancreaticodudenectomy
PDAC	pancreatic ductal adenocarcinoma
PE + AR	pancreatectomy with arterial resection
PFS	progression-free survival
PHDAC, PHC	pancreatic head ductal adenocarcinoma, pancreatic head cancer
POPF	postoperative pancreatic fistula
RGEA	right gastro-epiploic artery
SA	splenic artery
SMA	superior mesenteric artery
SPDP SVR	spleen-preserving distal pancreatectomy with splenic vessels resection (Sutherland-Warshaw procedure)
SPDP SVP	spleen-preserving distal pancreatectomy with splenic vessels preservation (Mallet-Guy–Kimura procedure)
SP	spleen preserving
SVR	splenic vessels resection
SVP	splenic vessels preservation
TP	total pancreatectomy
VBF = BFI	volumetric blood flow = blood flow intensity
