# Supplementary material for: Three Types of Collateral Arterial Supply to the Spleen After Spleen-Preserving Distal Pancreatectomies with Splenic Vessels Resection—How to Use This Knowledge for Organ(s) Preservation in Locally Advanced and Borderline Resectable Pancreatic Head Cancers Surgery—Hemodynamic, Surgical and Oncological Outcomes of 134 Spleen-Preserving Pancreatectomies
Source: Cancers (Basel). 2026 May 21;18(10):1675. doi: 10.3390/cancers18101675 (PMC13204045; doi:10.3390/cancers18101675)
Supplement: Supplementary file 1 [file cancers-18-01675-s001.zip › File S1. Report of the Ethics Committee. Egorov et al. .pdf]

ILYINSKAYA
HOSPITAL

Integrated healthcare practice

Ilyinskaya Hospital JSC
Build. 2 2 Rublevskoye Predmestye St.,
vill. Glukhovo, Krasnogorskiy region,
MA, Russia, 143421
www.ihospital.ru

+7 (495) 645-33-77
ih@ihospital.ru
License for medical activities
LO41-01162-50/00377253 from December 24, 2020
PSRN 1157746997374

Ilyinskaya Hospital Ethics Committee

Report of the Ethics Committee (extract)
№ 12-02-SA/2021 of May 14, 2021

Committee members
Alexei Zhivov, MD, PhD (Chairperson of the Committee)
Evgeny Dzhenzhera, MD
Vyacheslav Egorov, MD, PhD
Dmitry Shchekochikhin, MD
Mikhail Vyborniy, MD
Vadim Sizov, MD
Sergey Terekhin, MD
Olga Durnetsova, MD

Committee Secretary
Grigory Bolshakov
Anastasia Los

Committee meeting agenda
Ethical approval of research study:

Retrospective study of safety and oncological outcomes of the pancreatectomies for locally advanced pancreatic malignancies, associated with arterial and venous resections.

Decision point (consensus decision):
Ilyinskaya Hospital Ethics Committee approves the implementation of the above research work and recognizes the study as ethically correct.

Research Supervisor: Vyacheslav Egorov, MD, PhD.

Alexei Zhivov, MD, PhD
Chairperson of the Ethics Committee
