# Supplementary material for: Three Types of Collateral Arterial Supply to the Spleen After Spleen-Preserving Distal Pancreatectomies with Splenic Vessels Resection—How to Use This Knowledge for Organ(s) Preservation in Locally Advanced and Borderline Resectable Pancreatic Head Cancers Surgery—Hemodynamic, Surgical and Oncological Outcomes of 134 Spleen-Preserving Pancreatectomies
Source: Cancers (Basel). 2026 May 21;18(10):1675. doi: 10.3390/cancers18101675 (PMC13204045; doi:10.3390/cancers18101675)
Supplement: Supplementary file 1 [file cancers-18-01675-s001.zip › File S2. Consent. Egorov et al.pdf]

Machine Translated by Google

Joint-Stock Company "Ilyinskaya Hospital"

143421, Moscow region, Krasnogorsk urban district, Glukhovo village, Rublevskoye Predmestye street, 2, building 2

Tel.: +7 (495) 645-33-77
ih@ihospital.ru
License L041-01162-50/00377253 of December 24, 2020

Voluntary informed consent for performance

(compiled in accordance with Articles 20, 22 of the Federal Law of 21.11.2011 No. 323-FZ “On the Fundamentals of Health Protection of Citizens in the Russian Federation”).

Informed consent must be prepared and signed by the healthcare professional (physician) directly involved in the patient's treatment. If the patient undergoes surgery/invasive manipulation or procedure, the informed consent should, if possible, be prepared and signed by the physician (surgeon) performing the procedure/procedure.

The healthcare professional explains the contents of this informed consent to the patient in a language accessible to the patient, with minimal use of complex and incomprehensible medical terms, and, if necessary, with the use of graphic and video illustrations, answers all patient questions in detail, and ensures that the patient understands the information conveyed to him.

1. This informed consent is prepared for the patient:

2. The physician who prepared this informed consent and his role in the patient’s treatment:

3. Information about the upcoming operation/procedure/manipulation.

The physician who prepared this informed consent and his role in the patient's care

3.1. Diagnosis

3.2. Internationally accepted name of the operation/medical procedure/ manipulation

3.3. Indications for surgery/medical procedure/manipulation

3.4. Planned anesthesia

3.5. Description/method of performing the operation/medical procedure/manipulation

3.6. Estimated duration in hours

3.7. Single-stage or multi-stage operation

3.8. The expected benefit from performing this operation/medical procedure/manipulation, its effectiveness in treating the patient’s existing disease

3.9. Expected risks, complications, side effects, relapses after this operation/medical procedure/ manipulation and their frequency in % (if known)

Machine Translated by Google

3.10. Expected risks, complications, side effects, relapses as a result of refusal to perform this operation/medical procedure/manipulation and their frequency in % (if known)

3.11. Alternative diagnostic and treatment methods to this operation/medical procedure/manipulation. Comparative characteristics of alternative and the proposed method.

3.12. Features of the course of the perioperative/periprocedural/perimanipulative period

4. I, (FULL NAME), the operating surgeon for patient (FULL NAME, EMR number), have, through a thorough interview and detailed conversation, verified that, as a result of my comprehensive information regarding the upcoming surgery/medical procedure/manipulation, the patient (FULL NAME) has understood the reasons for and indications for it, the technique, the expected benefits and effectiveness, the possible risks and complications, the likelihood of disease recurrence and, therefore, the need for repeat or additional medical procedures, the consequences of refusing the surgery/procedure/manipulation, as well as alternatives to the proposed surgery/medical procedure/manipulation. I have also thoroughly explained the specifics of the postoperative period and follow-up.

5. I, FULL NAME, date of birth, having clarified and considered the information provided to me, consider it to be exhaustive, reliable and sufficient for making an informed voluntary decision.

6. I, FULL NAME, Date of Birth, acknowledge that complications and recurrences of the disease described in this consent form may occur even with proper and high-quality performance of the surgery/medical procedure/manipulation. The likelihood and occurrence of such an adverse event depend not only on the qualifications, skills, and actions of the physician, but also on patient factors, including, but not limited to, concomitant diseases and conditions prior to the surgery/medical procedure/manipulation, nutritional status and metabolism, individual wound healing characteristics, the presence of chronic infections, including hospital-acquired ones, previously acquired in other medical institutions, allergic reactions, and individual intolerances. All of these adverse factors cannot always be properly assessed, controlled, and mitigated with the current level of medical knowledge. I also understand that if complications from the surgery/medical procedure/manipulation develop or the disease recur, additional treatment may be required, which will be paid for separately.

7. I, FULL NAME, date of birth, consciously and voluntarily entrust the operating surgeon (FULL NAME) and give my voluntary, informed consent to perform the surgery in accordance with the terms and conditions described in this consent. I also entrust the attending physician/operating surgeon to make decisions during the surgery regarding changes to its course and/or technique, if necessary, to use all of their knowledge, skills, technical means, necessary medications and consumables, and to undertake other medical and organizational actions to achieve the highest positive effect and the best possible outcome for the patient's health, and, if necessary, to involve other specialists to conduct a consultation or provide direct assistance during the surgery/medical procedure/ manipulation. I also understand and acknowledge that changes to the original plan and course of the surgery/invasive manipulation/procedure may entail an increase in the cost of treatment and require additional payment.

8. I, FULL NAME, DATE OF BIRTH, confirm that I have provided the doctor and staff of the Ilyinskaya Hospital with accurate information regarding my absence, at the time of signing this Informed Voluntary Consent, of the COVID-19 coronavirus infection, symptoms of acute respiratory viral infections (including latent ones), and my absence of any contact with persons in quarantine and/or mandatory self-isolation.

Machine Translated by Google

I confirm that I am not (am not required to be) in quarantine and/or under mandatory self-isolation, in accordance with the current legislation (including by-laws) of the Russian Federation.

I understand that my presence of COVID-19 coronavirus infection (both at the time of my stay, treatment, diagnosis, and prophylaxis, and after their onset) precludes my stay, treatment, diagnosis, and prophylaxis at Ilyinskaya Hospital; and if this infection is detected, I will be transferred to the appropriate infectious disease facility.

I fully understand that COVID-19 coronavirus infection may have a delayed onset of symptoms, and that my COVID-19 test result at the time of signing this Informed Voluntary Consent may be negative, indicating that I may already be a carrier (transmitter) of the infection. Therefore, my subsequent detection of COVID-19 coronavirus infection and a positive test result for the specified strain of infection during (or after) my stay, treatment, diagnosis, and prophylaxis at Ilyinskaya Hospital cannot be considered objective confirmation of my infection at Ilyinskaya Hospital (from the doctor or other Ilyinskaya Hospital staff) and cannot serve as grounds for any property or non-property claims against Ilyinskaya Hospital, the doctor, or any other Ilyinskaya Hospital staff.

I hereby confirm and fully understand that the provision of inaccurate and/or false information, as well as the distortion of the information provided, may be grounds for bringing me to administrative and/or criminal liability by the competent authorities in accordance with the procedures established by the legislation of the Russian Federation.

I confirm that I have provided the doctor with all information regarding my health, lifestyle, occupation (including work in hazardous industries, professional sports, etc.), living conditions, allergies, habits, and any other nuances and characteristics that may have a direct or indirect impact on my health.

I fully understand that the circumstances and conditions I have described have/may have a significant impact on my health, the timing, conditions, procedures, and risks of medical interventions, the possibility of using alternative treatments, the consequences of refusing the proposed medical intervention, the period and timeframe of rehabilitation and recovery, recommendations and restrictions after medical interventions (including surgeries, punctures, etc.), and may significantly influence the prescription of treatment (adjustment of previously prescribed treatment) and subsequent monitoring. All facts I have provided will be taken into account by the physician and other medical staff of JSC Ilyinskaya Hospital when providing me with medical care.

I have been warned and fully understand that in order to achieve the maximum effect from treatment and prevention, reduce the risk of side effects, complications, including from unwanted drug interactions, in the interests of my safety, I have informed (I undertake to inform during the course of providing me with medical care at JSC Ilyinskaya Hospital) the attending physician about all, without exception, medications, food and biologically active supplements (BAS), hormonal drugs, other doping and stimulants, as well as narcotic and psychotropic drugs that I take, including in connection with sports and professional activities, as well as according to the prescriptions and recommendations of other doctors or consultants, or at my own discretion.

I fully understand that concealing, distorting, or providing incomplete information and facts about my health, the factors influencing it, my lifestyle, occupation, medications and non-medicinal products I take, etc., may have a significant (in some cases, critical) impact on my health. I fully understand that concealing, distorting, or providing incomplete information may (directly or indirectly) cause harm to my health, my health may worsen, and the expected treatment outcome may not be achieved. However, such developments will not be the result of fault (including direct and/or indirect intent; negligence and/or carelessness) on the part of the attending physician or other staff of Ilyinskaya Hospital, including administrative personnel.

Signature of the patient (legal representative/representative by power of attorney) _____________

Machine Translated by Google

Signature of the operating surgeon ________________

Signature of the attending physician ________________

April 2, 2026
