# Supplementary material for: Three Types of Collateral Arterial Supply to the Spleen After Spleen-Preserving Distal Pancreatectomies with Splenic Vessels Resection—How to Use This Knowledge for Organ(s) Preservation in Locally Advanced and Borderline Resectable Pancreatic Head Cancers Surgery—Hemodynamic, Surgical and Oncological Outcomes of 134 Spleen-Preserving Pancreatectomies
Source: Cancers (Basel). 2026 May 21;18(10):1675. doi: 10.3390/cancers18101675 (PMC13204045; doi:10.3390/cancers18101675)
Supplement: Supplementary file 1 [file cancers-18-01675-s001.zip › File S3. PROCESS 2023 guidelines checklist Egorov et al. .pdf]

PROCESS 2020 guidelines and the proposed version of PROCESS 2023 guidelines.

| Topic | Item | PROCESS 2020 | Proposed PROCESS 2023 |
|---|---|---|---|
| Title | 1 | The phrase ‘case series’ and the area of focus should appear in the title (e.g. patient population, diagnosis, intervention or outcome). | The phrase ‘case series’ is included<br>• The focus of the research study is mentioned (e.g. patient population, setting, diagnosis, intervention, outcome etc.) |
| Keywords | 2 | Include three to six keywords that identify what is covered in the case series (e.g. patient population, diagnosis, intervention or outcome).<br>• Include ‘case series’ as one of the keywords. | Include three to six keywords that identify what is covered in the case series (e.g. patient population, setting, diagnosis, intervention, outcome etc.)<br>• Include ‘case series’ as one of the keywords<br>• Include the surgical subspeciality the case series pertains to as a keyword |
| Abstract | 3a | Introduction and Importance<br>• Describe what is unique or educational.<br>• What is the overarching theme of the case series? | Introduction – briefly describe:<br>• Background<br>• Scientific rationale for this study<br>• Overarching theme of the case series<br>• Aims and objectives |
| | 3b | Methods<br>• Describe what was done, how and when was it done and by whom. | Methods – briefly describe:<br>• Sample size<br>• Timeframe of research<br>• Characteristics of study design (e.g. prospective/retrospective, single-/multicentre, informal/formal, consecutive/nonconsecutive, exposure-/outcome-based sampling, clinical/population-based etc.) |
| | 3c | Outcomes<br>• Describe the outcomes of the intervention and management strategy. | Results – briefly describe:<br>• Outcomes of the intervention/management strategy |
| | 3d | Conclusion<br>• Describe the take-home message(s), including what has been learnt?<br>• How will this impact future clinical practice? | Conclusion – briefly describe:<br>• Key findings and take-home messages<br>• Impact on future clinical practice<br>• Direction of future research |
| | 3e | | Present a structured abstract<br>• Informal case series – introduction, case presentations (brief description of each case) and discussion/conclusion<br>• Formal case series – introduction, methods, results and discussion/conclusion |

| Topic | Item | PROCESS 2020 | Proposed PROCESS 2023 |
|---|---|---|---|
| Highlights | 4 | | Convey the key findings of the research study in 3–5 bullet points |
| Introduction | 5 | Describe the background of the case series and specify the overarching theme (e.g. common disease, intervention, or outcome).<br>• The introduction should explain what is unique or educational about the case series.<br>• Relevant scientific literature should be referenced.<br>• Introduction should be 1–2 paragraphs in length. | Introduction – comprehensively describe:<br>• Relevant background and scientific rationale for case series with reference to key scientific literature<br>• Overarching theme (e.g. common patient population, setting, diagnosis, intervention, outcome etc.)<br>• Aims and objectives |
| Methods | 6a | Registration<br>• State the research registry number in accordance with the Declaration of Helsinki - "Every research study involving human subjects must be registered in a publicly accessible database". This can be obtained from, for example, ResearchRegistry.com, ClinicalTrials.gov, or ISRCTN.<br>• If a protocol already exists, state the corresponding registration number and access directions (e.g. website or journal, and include a hyperlink that is publicly accessible). It must be written in the English language. | Registration<br>• In accordance with the Declaration of Helsinki*, state the research registration number and where it was registered, with a hyperlink to the registry entry (this can be obtained from ResearchRegistry.com, ClinicalTrials.gov, ISRCTN etc.)<br>• All retrospective studies should be registered before submission; it should be stated that the research was retrospectively registered<br>• *"Every research study involving human subjects must be registered in a publicly accessible database before recruitment of the first subject"* |
| | 6b | | Protocol<br>• If a protocol exists, state the corresponding registration number and access directions (e.g. website or journal, and include a hyperlink that is publicly accessible).<br>• It must be written in the English language. |
| | 6c | | Ethical approval<br>• State whether ethical approval was needed or not, with reason(s)<br>• If appropriate, state name of body giving ethical approval and approval number |
| | 6d | Study Design<br>• State that the study is a case series.<br>• State whether the case series is: (1) prospective/retrospective, (2) single/multicentre, and if (3) cases are consecutive/nonconsecutive. | Study design<br>• State that the study is a case series<br>• Describe key characteristics of study design (e.g. prospective/retrospective, single-/multicentre, informal/formal, consecutive/nonconsecutive, exposure- |

| Topic | Item | PROCESS 2020 | Proposed PROCESS 2023 |
|---|---|---|---|
| | | | /outcome-based sampling, clinical/population-based etc.) |
| | 6e | Settings and Time-Frames<br>• Describe the setting(s) in which the patient was managed (e.g. research institution, teaching/district general hospital, community, or private practice).<br>• Document any relevant dates (e.g. recruitment, intervention, follow-up, and data collection time-frames). | Setting and timeframe – comprehensively describe:<br>• Geographical location<br>• Nature of setting(s) where the patient was managed (e.g. primary/secondary/tertiary care setting, district general hospital/teaching hospital, public/private, low-resource setting etc.)<br>• Relevant dates (e.g. recruitment, intervention, follow-up, data collection etc.) |
| | 6f | Participants<br>• Describe the relevant characteristics (e.g. demographics, comorbidities, tumour staging, smoking status) and if relevant, exposure(s) of the participants.<br>• Describe the method of participant recruitment, if relevant.<br>• State any subsequent inclusion or exclusion criteria, and how the participants were selected.<br>• Methods used to ensure the de-identification of patient information. | Participants – comprehensively describe:<br>• Relevant participant characteristics (e.g. demographics, comorbidities, ASA score, severity of surgery, urgency of surgery, smoking status, tumour staging etc.) and if relevant, exposure(s) of the participants (e.g. COVID-19)<br>• Subsequent inclusion and exclusion criteria with clear definitions<br>• Approach to selecting patients (e.g. consecutive/nonconsecutive, exposure-/outcome-based, formal/informal etc.)<br>• Methods used to ensure de-identification of patient information |
| | 6g | | Recruitment – comprehensively describe:<br>• Sources of recruitment (e.g. physician referral, electronic health record etc.)<br>• Any monetary incentivisation of patients for recruitment and retention should be declared; clarify the nature of any incentives provided |
| | 6h | Preintervention Patient Optimisation<br>• Lifestyle (e.g. weight loss).<br>• Medication review (e.g. anticoagulation, oral hypoglycemics/insulin).<br>• Presurgical stabilisation/preparation (e.g. treating hypothermia/hypovolemia/hypotension, ICU care for sepsis, nil by mouth, or enema).<br>• Other (e.g. psychological support). | Preintervention patient optimisation:<br>• Lifestyle (e.g. weight loss, nutritional support, exercise, smoking cessation etc.)<br>• Medication review (e.g. anticoagulation, oral hypoglycemics, insulin, oral contraceptive pill etc.)<br>• Presurgical stabilisation/preparation (e.g. treating hypothermia/-volemia/-tension, ICU care, nil by mouth, bowel preparation etc.)<br>• Other (e.g. psychological support, preoperative education/counselling etc.) |

| Topic | Item | PROCESS 2020 | Proposed PROCESS 2023 |
|---|---|---|---|
| | 6i | Interventions<br>• Describe the type(s) of intervention(s) used (e.g. pharmacological, surgical, physiotherapy, psychological, preventative).<br>• Describe any concurrent treatments (e.g. antibiotics, analgesia, antiemetics, venous thromboembolism prophylaxis). | Interventions – comprehensively describe:<br>• Type of intervention (e.g. pharmacological, surgical, physiotherapy, psychological etc.)<br>• Aim of intervention (preventative/therapeutic)<br>• Concurrent treatments (e.g. antibiotics, analgesia, antiemetics, venous thromboembolism prophylaxis etc.) |
| | 6j | Intervention Details<br>• Describe the rationale behind the treatment offered, how it was performed and time to intervention.<br>• For pharmacological therapies, include information on the formulation, dosage, strength, route, and duration.<br>• For surgery, include details such as anaesthesia, patient position, preparation used, use of other relevant equipment, sutures, devices, and surgical stage.<br>• The degree of novelty for a surgical technique/device should be mentioned (e.g. 'first in human' or 'first in this context').<br>• Medical devices should have manufacturer and model specifically mentioned. | Intervention specifics – comprehensively describe:<br>• Rationale for the treatment offered<br>• Techniques involved in the administration of the intervention<br>• Time to intervention<br>• For pharmacological therapies, include details such as formulation, dosage, strength, route and duration<br>• For surgical intervention, include details on anaesthesia, patient positioning, preparation used, equipment needed, devices, sutures, surgical stage etc.<br>• Degree of novelty of surgical technique/device (e.g. 'first in human' or 'first in this context')<br>• Manufacturer and model of any medical devices used |
| | 6k | Operator Details<br>• Where applicable, include operator experience and position on the learning curve, any relevant training, and specialisation (e.g. 'junior trainee with 3 years of surgical specialty training in Plastic Surgery and seven similar cases completed previously under direct supervision'). | Operator details – comprehensively describe:<br>• Relevant training, specialisation and operator's experience (e.g. average number of the relevant procedures performed annually, independent, needs direct/indirect supervision etc.)<br>• Learning curve for technique<br>• Requirement for additional training |
| | 6l | Quality Control<br>• What measures were taken to reduce inter- or intra-operator/operation variation, to ensure quality, and to maintain consistency between cases (e.g. independent observers, lymph node counts, standard surgical technique).<br>• State any specific disparities between cases. | Quality control – comprehensively describe:<br>• Measures taken to reduce inter- or intra-operator/operation variation, ensure quality and maintain consistency between cases (e.g. independent observers, lymph node counts, standard surgical technique etc.)<br>• Any specific disparities between cases |
| | 6m | Follow-Up<br>• When (e.g. how long after discharge, frequency, | Postoperative care and follow-up – comprehensively describe: |

| Topic | Item | PROCESS 2020 | Proposed PROCESS 2023 |
|---|---|---|---|
| | | maximum follow-up length at the time of submission).<br>• Where (e.g. home via video consultation, primary care, secondary care).<br>• How (e.g. telephone consultation, clinical examination, blood tests, imaging).<br>• Any specific long-term surveillance requirements (e.g. imaging surveillance of endovascular aneurysm repair or clinical exam/ultrasound of regional lymph nodes for skin cancer).<br>• Any specific postoperative instructions (e.g. postoperative medications, targeted physiotherapy, psychological therapy).<br>• State if any participants were lost to follow-up and why. | • Postoperative care (e.g. patient education, postoperative medications, early mobilisation, targeted physiotherapy, early enteral nutrition, early removal of catheters/drains, psychological therapy etc.)<br>• Follow-up time-frames (e.g. first follow-up postdischarge, follow-up duration at the time of submission etc.) and frequency<br>• Follow-up setting (e.g. home via phone/video consultation, primary care, secondary care etc.)<br>• Follow-up method (e.g. history, clinical examination, blood tests, imaging etc.)<br>• Follow-up personnel (e.g. operating surgeon)<br>• Any specific long-term surveillance requirements (e.g. imaging surveillance of endovascular aneurysm repair, clinical/ultrasound examination of regional lymph nodes for skin cancer etc.)<br>• State if any participants were lost to follow-up and why |
| Results | 7a | Participants<br>• Please state the number of patients involved, the patient characteristics (e.g. demographics, comorbidities, smoking status, and if applicable, tumour staging (e.g. TNM)). | Participants – comprehensively describe:<br>• Number of patients involved<br>• Patient characteristics (e.g. demographics, comorbidities, ASA score, severity of surgery, urgency of surgery, smoking status, tumour staging etc.) and if relevant, exposure(s) of the participants<br>• Include table showing baseline patient characteristics |
| | 7b | Deviation from the Initial Management Plan<br>• State if there were any changes in the planned intervention(s) (e.g. what was changed and why).<br>• Please include a suitable schematic diagram if appropriate. | Deviation from the initial management plan – comprehensively describe:<br>• Any changes to the planned intervention with rationale<br>• If appropriate, include a suitable schematic diagram<br>• |
| | 7c | Outcomes and Follow-Up<br>• Expected versus attained clinical outcome as assessed by the clinician. Reference literature used to inform expected outcomes.<br>• When appropriate, include patient-reported measures (e.g. questionnaires including quality-of-life scales). | Outcomes and follow-up – comprehensively describe:<br>• Expected versus attained clinician assessed outcome, providing reference to scientific literature used to inform expected outcomes (e.g. core outcome set)<br>• If appropriate, include patient-reported outcomes (e.g. quality-of-life) |

| Topic | Item | PROCESS 2020 | Proposed PROCESS 2023 |
|---|---|---|---|
| | | • Describe and explain the percentage of patients lost to follow-up. | • Percentage of patients lost to follow-up with rationale |
| | 7d | Intervention Adherence and Compliance<br>• Where relevant, detail how well the patient adhered to and tolerated the advice provided (e.g. avoiding heavy lifting for abdominal surgery, or tolerance of chemotherapy and pharmacological agents).<br>• Explain how adherence and tolerance were measured. | Intervention adherence and compliance – comprehensively describe:<br>• Assessment of patient's adherence and tolerability of intervention and postoperative instructions (e.g. avoiding heavy lifting/strenuous activity, tolerance of chemotherapy/pharmacological agents etc.)<br>• Impact on long-term applicability of intervention in clinical practice |
| | 7e | Complications and Adverse Events<br>• Precautionary measures taken to prevent complications (e.g. antibiotic or venous thromboembolism prophylaxis).<br>• All complications and adverse or unanticipated events should be described in detail and ideally categorised in accordance with the Clavien–Dindo Classification (e.g. blood loss, length of operative time, wound complications, re-exploration or revision surgery, impact on length of stay).<br>• If relevant, was the complication reported to the relevant national agency or pharmaceutical company.<br>• Specify the duration of time between completion of the intervention and discharge, and whether this was within the expected timeframe (if not, why not).<br>• Where applicable, the 30-day postoperative and long-term morbidity/mortality may need to be specified.<br>• State if there were no complications or adverse outcomes | Complications and adverse events – comprehensively describe:<br>• Precautionary measures taken to prevent complications (e.g. antibiotic/venous thromboembolism prophylaxis)<br>• Complications and adverse events (e.g. blood loss, wound infection, deep vein thrombosis, pulmonary embolism etc.), categorised in accordance with the Clavien–Dindo classification<br>• Timing of adverse events<br>• Mitigation for adverse events (e.g. blood transfusion, wound care, re-exploration/revision surgery etc.)<br>• If appropriate, whether complications or adverse events were discussed locally (e.g. morbidity and mortality meetings)<br>• If appropriate, whether complications or adverse events were reported to the relevant national agency or pharmaceutical company<br>• Specify time to discharge following completion of intervention and whether this was within the expected timeframe or not (if not, why not)<br>• Where applicable, specify the 30-day postoperative and long-term morbidity/mortality<br>• State if there were no complications or adverse events |
| Discussion | 8a | Summarise the key results. | Key results–comprehensively describe:<br>• Key results with relevant raw data<br>• Include table showing key results |
| | 8b | Relevant Literature and Placing the Results in Context<br>• Include a discussion of the relevant literature and, if | Scientific context and implications–comprehensively describe: |

| Topic | Item | PROCESS 2020 | Proposed PROCESS 2023 |
|---|---|---|---|
| | | appropriate, similar published studies.<br>• Describe the implications for clinical practice guidelines (e.g. NICE) and any relevant hypotheses generated | • Relevant literature and if appropriate, similar published studies<br>• Implications for clinical practice and guidelines (e.g. NICE)<br>• Comparison to current gold standard of care<br>• Relevant hypothesis generation |
| | 8c | Strengths<br>• Describe the relevant strengths of the study.<br>• Detail any multidisciplinary or cross-speciality relevance.<br>• Weaknesses and Limitations<br>• Describe the relevant weaknesses or limitations of the study.<br>• For novel techniques or devices, outline any contraindications and alternatives, potential risks and possible complications if applied to a larger population | Strengths–comprehensively describe:<br>• Strengths of the study<br>• Any multidisciplinary or cross-speciality relevance |
| | 8d | | Weaknesses and limitations – comprehensively describe:<br>• Weaknesses and limitations of the study, with potential impact on results and their interpretation<br>• Deviations from protocol, with reasons<br>• For novel techniques or devices, outline any contraindications/alternatives and potential risks/complications if applied to a larger population |
| | 8e | Directions for Future Research<br>• State how the methodology and findings discussed can impact future research and clinical practice. Describe the questions that have arisen as a result of this study.<br>• State the alternative study design(s) best suited to address these questions | Directions for future research – comprehensively describe:<br>• Impact on future research and clinical practice<br>• Questions that have arisen as a result of the study<br>• Alternative study design(s) best suited to address these questions |
| | 8f | | Cost – comprehensively describe:<br>• Cost of intervention<br>• Justify cost if intervention more expensive than current gold standard of care<br>• Any cheaper alternatives |
| Conclusions | 9a | Key Conclusions<br>• Outline the key conclusions from this study | Key conclusions<br>• Outline the key conclusions from this study |

| Topic | Item | PROCESS 2020 | Proposed PROCESS 2023 |
|---|---|---|---|
| | 9b | Rationale<br>• Ensure that any of the conclusions made are supported by a strong rationale | Rationale<br>• Explain the rationale behind those conclusions |
| | 9c | Future Work<br>• Briefly discuss any questions arisen from this study and any differences in approach to patient diagnosis or management which the authors might adopt in future similar studies | Future work – briefly describe:<br>• Any questions arisen from the study<br>• Any differences in approach to patient diagnosis or management which authors might adopt in future similar studies |
| Patient and/or Carer Perspective | 10 | Where appropriate, the patients should be given the opportunity to share their perspective on the intervention(s) they received (e.g. sharing quotes from a consented, anonymised interview, or questionnaire) | Where appropriate, the patients should be given the opportunity to share their perspective on the intervention(s) they received (e.g. sharing quotes from a consented, anonymised interview or questionnaire) |
| Informed Consent | 11 | The authors must provide evidence of consent, where applicable, and if requested by the journal.<br>• State the method of consent at the end of the article (e.g. verbal or written).<br>• If not provided by the patients, explain why (e.g. death of patient and consent provided by next of kin). If the patients or family members were untraceable then document the tracing efforts undertaken | The authors must provide evidence of consent, where applicable, and if requested by the journal<br>• State the method of consent at the end of the article (e.g. verbal or written)<br>• If not provided by the patients, explain why (e.g. death of patient and consent provided by next of kin). If the patients or family members were untraceable then document the tracing efforts undertaken |
| Additional Information | 12a | State any conflicts of interest | State any conflicts of interest |
| | 12b | State any sources of funding | State any sources of funding (e.g. grant details)<br>• Role of funder |
| | 12c | Other Relevant Disclosures<br>• Please state any author contributions, acknowledgements, and where required, institutional review board and ethical committee approval.<br>• Disclose whether the case has been presented at a conference or regional meeting | Other relevant disclosures<br>• State any author contributions and acknowledgements<br>• If appropriate, give details of institutional review board and ethical committee approval<br>• Disclose whether the case has been presented at a conference or regional meeting |
| Clinical Images and Videos | 13 | Where relevant and available, include clinical images to help demonstrate the cases pre-, peri-, and postintervention (e.g. radiological, histopathological, patient photographs, intraoperative images).<br>• Where relevant and available, include a link (e.g. | Where relevant and available, include clinical images to help demonstrate the cases pre-, peri- and postintervention (e.g. radiological, histopathological, patient photographs, intraoperative images etc.) |

| Topic | Item | PROCESS 2020 | Proposed PROCESS 2023 |
| --- | --- | --- | --- |
| | | Google Drive, YouTube) to the narrated operative video to highlight specific techniques or operative findings.<br>• Ensure all media files are appropriately captioned and indicate points of interest to allow for easy interpretation | • Where relevant and available, include a link (e.g. Google Drive, YouTube etc.) to the narrated operative video to highlight specific techniques or operative findings<br>• Ensure all media files are appropriately captioned and indicate points of interest to allow for easy interpretation |
| Referencing the Checklist | 14 | Include reference to the PROCESS 2020 publication by stating: 'This case series has been reported in line with the PROCESS Guideline' at the end of the methods section (and include citation in the references section) | Include reference to the PROCESS 2023 publication by stating: 'This case series has been reported in line with the PROCESS Guideline' at the end of the methods section and include citation in the references section |
