# Supplementary material for: Three Types of Collateral Arterial Supply to the Spleen After Spleen-Preserving Distal Pancreatectomies with Splenic Vessels Resection—How to Use This Knowledge for Organ(s) Preservation in Locally Advanced and Borderline Resectable Pancreatic Head Cancers Surgery—Hemodynamic, Surgical and Oncological Outcomes of 134 Spleen-Preserving Pancreatectomies
Source: Cancers (Basel). 2026 May 21;18(10):1675. doi: 10.3390/cancers18101675 (PMC13204045; doi:10.3390/cancers18101675)
Supplement: Supplementary file 1 [file cancers-18-01675-s001.zip › File S4. STROCSS 2024 guidelines checklist Egorov et al.pdf]

Table 1

The STROCSS 2021 guidelines and the proposed version of the STROCSS 2024 guidelines.

Comparison of STROCSS 2021 and proposed STROCSS 2024

STROCSS 2021			Proposed STROCSS 2024		
**Topic**	**Item**	**Guideline**	**Topic**	**Item**	**Guideline**
Title	1	Title<br>The word cohort or cross-sectional or case–control is included*<br>Temporal design of study is stated (e.g. retrospective or prospective)<br>The focus of the research study is mentioned (e.g. population, setting, disease, exposure/intervention, outcome etc.)<br>*STROCSS 2021 guidelines apply to cohort studies as well as other observational studies (e.g. cross-sectional, case–control etc.)	Title	1	*Title*<br>The word 'cohort' or 'cross-sectional' or 'case–control' is included*<br>Temporal design of the study is stated (e.g. retrospective or prospective)<br>The focus of the study is clearly stated (e.g. population, setting, disease, exposure/intervention, outcome, etc.)<br>**STROCSS 2024 guidelines apply to all observational studies (e.g. cohort, cross-sectional, case–control, etc.)*
				2	*Highlights*<br>Include three to five bullet points that summarise the key findings of the study<br>Provide a brief background to the study, the key results and clinical relevance
			Abstract	3a	*Structure*<br>Provide a structured abstract that includes the following headings:<br>1. Background<br>2. Methods<br>3. Results<br>4. Conclusions
Abstract	2a	Introduction - briefly describe:<br>Background<br>Scientific rationale for this study<br>Aims and objectives		3b	*Background*<br>*Briefly describe*:<br>Relevant context<br>Scientific rationale for this study<br>Aims and objectives
	2b	Methods - briefly describe:<br>Type of study design (e.g. cohort, case–control, cross-sectional etc.)<br>Other key elements of study design (e.g. retro-/prospective, single/multicentred etc.)<br>Patient populations and/or groups, including control group, if applicable<br>Exposure/interventions (e.g. type, operators, recipients, timeframes etc.)<br>Outcome measures - state primary and secondary outcome(s)		3c	*Methods*<br>*Briefly describe*:<br>Type of study design (e.g. cohort, case–control, cross-sectional etc.)<br>Specification of study design (e.g. retro-/prospective, single/multicentred etc.)<br>All patient groups involved, including control group, if applicable<br>Exposure/interventions (e.g. type, operators, recipients, dates and time frames etc.)<br>Outcome measures - explicitly state primary and secondary outcome(s), where appropriate<br>Statistical methods of assessment used, where applicable
	2c	Results - briefly describe:<br>Summary data with qualitative descriptions and statistical relevance, where appropriate		3d	*Results*<br>*Briefly describe*:<br>Summary data<br>Principal findings with qualitative descriptions<br>Statistical findings and their significance, where appropriate
	2d	Conclusion - briefly describe:<br>Key conclusions<br>Implications for clinical practice<br>Need for and direction of future research		3e	*Conclusion*<br>Describe key conclusions briefly<br>Refer to implications for clinical practice and public health<br>Describe the need for and direction of future research<br>Include a concise statement that encapsulates the significance of the research and its contribution to the field
			Keywords	4	*Keywords*<br>Include three to six keywords that identify what is covered in the study (e.g. patient population, diagnosis, or surgical intervention)<br>Include study type as a keyword (e.g. cohort study, cross-sectional study, case–control study etc.)<br>Include surgical speciality as one of the keywords<br>Include study location as one of the keywords
Introduction	3	Introduction - comprehensively describe:<br>Relevant background and scientific rationale for study, with reference to key literature<br>Research question and hypotheses, where appropriate<br>Aims and objectives	Introduction	5a	*Introduction*<br>*By referencing key literature throughout, comprehensively describe*:<br>Relevant background and scientific rationale for study<br>Aims and objectives<br>Research question and hypotheses, where appropriate<br>Potential impact of research on future clinical practice<br>Economic relevance of study to society
				5b	*Guideline citation*<br>At the end of the introduction, refer to the STROCSS 2024 publication by stating: 'This cohort/cross-sectional/case–control study has been reported in line with the STROCSS guidelines [*include citation*]'
Methods	4a	Registration<br>In accordance with the Declaration of Helsinki*, state the research registration number and where it was registered, with a hyperlink to the registry entry (this can be obtained from ResearchRegistry.com, ClinicaTrials.goc, ISRCTN etc.)<br>All retrospective studies should be registered before submission; it should be stated that the research was retrospectively registered<br>* *'Every research study involving human subjects must be registered in a publicly accessible database before recruitment of the first subject'*	Additional information	12a	*Registration*<br>In accordance with the Declaration of Helsinki*, state the unique research registration number and where it was registered, with a hyperlink to the registry entry *(this can be obtained from ResearchRegistry.com, ClinicalTrials.gov, ISRCTN etc.)*<br>*N.B. All retrospective studies should be registered before submission; it should be stated that the research was retrospectively registered.*<br>* *'Every research study involving human subjects must be registered in a publicly accessible database before recruitment of the first subject'*
	4b	Ethical approval<br>Reason(s) why ethical approval was needed<br>Name of the body giving ethical approval and approval number<br>Where ethical approval was not necessary, reason(s) are provided		12b	*Ethical approval*<br>Whether ethical approval was needed or not, stated explicitly<br>Reason(s) why ethical approval was/was not needed<br>Name of the body giving ethical approval and approval number
				12c	*Informed consent*<br>State explicitly whether informed consent was obtained, or not.<br>State reason(s) why informed consent was/was not obtained<br>State the nature of consent (e.g. verbal, written, digital/virtual)*<br>The authors must provide evidence of consent, where applicable, and if requested by the journal<br>Consent should be provided for both the original intervention/procedure and publication of the study<br>*If consent was not provided by the patient, explain why (e.g. death of the patient and consent provided by next of kin). If the patient or family members were untraceable, then document the tracing efforts undertaken
	4c	Protocol<br>Give details of protocol (a *priori* or otherwise) including how to access it (e.g. web address, protocol registration number etc.)<br>If published in a journal, cite and provide full reference		12d	*Protocol*<br>Give details of protocol (a *priori* or otherwise) including how to access it (e.g. web address, DOI etc.)<br>Give details of protocol registration (e.g. protocol registration number, protocol registry's name etc.)<br>If published in a journal, cite and provide a full reference

Rashid et al. International Journal of Surgery (2024)

Table 1

(Continued)

Comparison of STROCSS 2021 and proposed STROCSS 2024

STROCSS 2021			Proposed STROCSS 2024		
**Topic**	**Item**	**Guideline**	**Topic**	**Item**	**Guideline**
					If applicable, detail any amendments made to the original protocol, giving reasons why the changes were made
	4d	Patient and public involvement in research<br>Declare any patient and public involvement in research<br>State the stages of the research process where patients and the public were involved (e.g. patient recruitment, defining research outcomes, dissemination of results etc.) and describe the extent to which they were involved.			
	5a	Study design<br>State the type of study design used (e.g. cohort, cross-sectional, case–control etc.)<br>Describe other key elements of study design (e.g. retro-/ prospective, single/multicentred etc.)	Methods: Study Design	6a	*Study design*<br>State the type of study design (e.g. cohort, cross-sectional, case–control etc.)<br>Describe other key elements of study design (e.g. retro-/prospective, single/multi-centred etc.)<br>Specify the duration of the study, including start and end dates
	5b	Setting and timeframe of research - comprehensively describe<br>Geographical location<br>Nature of institution (e.g. primary/secondary/tertiary care setting, district general hospital/teaching hospital, public/private, low-resource setting etc.)<br>Dates (e.g. recruitment, exposure, follow-up, data collection etc.)		6b	*Setting and timeframe of research*<br>*Comprehensively describe*:<br>Specific geographical location<br>Nature of institution (e.g. primary/secondary/tertiary care setting, district general hospital/ teaching hospital, public/private, l- ow-resource setting etc.)<br>Timeline for study, including dates for recruitment, exposure, follow-up, data collection etc.<br>Any deviations from the initial study design plan or changes to the timeline during the research, with reasons and implications stated
	5c	Study groups<br>Total number of participants<br>Number of groups<br>Detail exposure/intervention allocated to each group<br>Number of participants in each group		6c	*Study groups*<br>Total number of participants<br>Number of groups<br>Number of participants in each group<br>Detail exposure/intervention allocated to each group<br>Inclusion and exclusion criteria with clear definitions
	5d	Subgroup analysis - comprehensively describe<br>Planned subgroup analyses<br>Methods used to examine subgroups and their interactions		6d	*Subgroup analysis*<br>*Comprehensively describe*:<br>How subgroups were defined<br>Planned subgroup analyses<br>Methods used to examine subgroups and their interactions
	6a	Participants - comprehensively describe<br>Inclusion and exclusion criteria with clear definitions<br>Sources of recruitment (e.g. physician referral, study website, social media, posters etc.)<br>Length, frequency and methods of follow-up (e.g. mail, telephone etc.)		6e	*Follow-up*<br>*If applicable, comprehensively describe*:<br>Time, length, frequency, location and methods of follow-up (e.g. mail, telephone, with whom etc.)<br>Any specific long-term surveillance requirements (e.g. imaging surveillance of endovascular aneurysm repair)<br>Any specific postoperative instructions (e.g. postoperative medications, targeted physiotherapy etc.)
	6b	Recruitment - comprehensively describe<br>Methods of recruitment to each patient group (e.g. all at once, in batches, continuously till desired sample size is reached etc.)<br>Any monetary incentivisation of patients for recruitment and retention should be declared; - clarify the nature of any incentives provided<br>Nature of informed consent (e.g. written, verbal etc.)<br>Period of recruitment	Methods: Participant Recruitment	7a	*Recruitment*<br>*Comprehensively describe*:<br>Period of recruitment<br>Methods of recruitment to each patient group (e.g. all at once, in batches, continuously till desired sample size is reached etc.)<br>Sources of recruitment (e.g. physician referral, study website, social media, posters etc.)<br>Any monetary/non-monetary incentivisation of participants to encourage involvement should be declared *(the nature of any incentives provided must be clarified)*<br>Any challenges encountered during the recruitment processes, including how they were addressed
	6c	Sample size - comprehensively describe<br>Analysis to determine optimal sample size for study accounting for population/effect size<br>Power calculations, where appropriate<br>Margin of error calculation		7b	*Sample size*<br>*Comprehensively describe*:<br>Analysis to determine optimal sample size for study accounting for population/effect size<br>Power calculations with justifications for chosen statistical power, where appropriate<br>Margin of error calculation<br>Any associated ethical considerations
Methods - Intervention and Considerations	7a	Preintervention considerations - comprehensively describe<br>Preoperative patient optimisation (e.g. weight loss, smoking cessation, glycaemic control etc.)<br>Preintervention treatment (e.g. medication review, bowel preparation, corrective hypothermia/-volemia/-tension, mitigating bleeding risk, ICU care etc.)	Methods: Intervention and Outcomes	8a	*Preintervention considerations*<br>*Comprehensively describe any preoperative patient optimisation*:<br>Lifestyle optimisation (e.g. weight loss, smoking cessation, glycaemic control etc.)<br>Medical optimisation (e.g. medication review, treating hypothermia/-volemia/-tension, ICU care etc.)<br>Procedural optimisation (e.g. nil by mouth, enema etc.)<br>Other (e.g. psychological support, physiotherapy etc.)
	7b	Intervention - comprehensively describe<br>Type of intervention and reasoning (e.g. pharmacological, surgical, physiotherapy, psychological etc.)<br>Aim of intervention (preventative/therapeutic)<br>Concurrent treatments (e.g. antibiotics, analgesia, antiemetics, VTE prophylaxis etc.)<br>Manufacturer and model details, where appropriate		8b	*Intervention*<br>*Comprehensively describe*:<br>Type of intervention and reasoning (e.g. pharmacological, surgical, physiotherapy, psychological etc.)<br>Aim of intervention (e.g. preventative/therapeutic)<br>Total cost of performing the intervention<br>Degree of novelty of intervention<br>Any learning required for intervention<br>Prevalence or frequency at which the intervention is performed<br>Concurrent treatments (e.g. antibiotics, analgesia, antiemetics, VTE prophylaxis etc.)<br>Manufacturer and model details, where appropriate
	7c	Intraintervention considerations - comprehensively describe<br>Details pertaining to administration of intervention (e.g. anaesthetic, positioning, location, preparation, equipment needed, devices, sutures, operative techniques, operative time etc.)<br>Details of pharmacological therapies used, including formulation, dosages, routes and durations<br>Figures and other media are used to illustrate		8c	*Intraintervention considerations*<br>*Using figures and other media to illustrate wherever appropriate, comprehensively describe*:<br>Details pertaining to administration of intervention (e.g. anaesthetic, positioning, location, preparation, equipment needed, devices, sutures, operative techniques, operative time etc.)<br>For pharmacological therapies, the formulation, dosages, routes, strength and durations<br>For surgery, any postoperative instruction (e.g. when to remove staples or sutures)<br>The degree of novelty for a surgical technique/device (e.g. 'first in human')
	7d	Operator details - comprehensively describe<br>Requirement for additional training<br>Learning curve for technique<br>Relevant training, specialisation and operator's experience (e.g. average number of the relevant procedures performed annually)		8d	*Operator details*<br>*Comprehensively describe*:<br>Requirement for additional training<br>Learning curve for technique, including how it was evaluated (e.g. number of cases required to reach a defined level of proficiency)<br>Relevant training, specialisation, and operator's experience (e.g. average number of the relevant procedures performed annually)<br>Any institutional support that was provided to operators to facilitate their training
				8e	*Setting of intervention*<br>*Comprehensively describe*:

Table 1

(Continued)

Comparison of STROCSS 2021 and proposed STROCSS 2024

STROCSS 2021			Proposed STROCSS 2024		
**Topic**	**Item**	**Guideline**	**Topic**	**Item**	**Guideline**
					Setting in which the intervention was performed<br>Level of experience the centre has in performing the intervention
	7e	Quality control - comprehensively describe<br>Measures taken to reduce interoperator variability<br>Measures taken to ensure consistency in other aspects of intervention delivery<br>Measures taken to ensure quality in intervention delivery		8f	*Quality control*<br>*Comprehensively describe*:<br>Measures taken to reduce interoperator variability (e.g. regular team meetings, calibration exercises)<br>Measures taken to ensure consistency in other aspects of intervention delivery (e.g. data collection)<br>Measures taken to ensure quality in intervention delivery
	7f	Postintervention considerations - comprehensively describe<br>Postoperative instructions (e.g. avoid heavy lifting) and care<br>Follow-up measures<br>Future surveillance requirements (e.g. blood tests, imaging etc.)		8g	*Postintervention considerations*<br>*Comprehensively describe*:<br>Postoperative instructions and care (e.g. avoid heavy lifting, dietary restrictions etc.)<br>Follow-up measures<br>Future surveillance requirements (e.g. blood tests, imaging etc.)<br>How patient engagement with postintervention instructions will be encouraged and monitored<br>If applicable, the criteria for patient discharge from the medical facility
	8	Outcomes - comprehensively describe<br>Primary outcomes, including validation, where applicable<br>Secondary outcomes, where appropriate<br>Definition of outcomes<br>If any validated outcome measurement tools are used, give full reference<br>Follow-up period for outcome assessment, divided by group		8h	Definition of outcomes<br>Define primary outcomes, including validation with full reference to relevant studies, where applicable<br>Define secondary outcomes, where appropriate<br>Describe methods or instruments used to measure each outcome, with full reference given if validated<br>Describe follow-up period for outcome assessment, divided by group
	9	Statistics - comprehensively describe<br>Statistical tests and statistical package(s)/software used<br>Confounders and their control, if known<br>Analysis approach (e.g. intention to treat/per protocol)<br>Any subgroup analyses<br>Level of statistical significance		8i	*Statistics*<br>*Comprehensively describe*:<br>Statistical tests and statistical package(s)/software used<br>Rationale behind the statistical tests/software of choice<br>Confounders and their control, if known<br>Analysis approach (e.g. intention to treat/per protocol)<br>Any subgroup analyses<br>Level of statistical significance<br>How the results of the statistical analyses are presented (e.g. *P* values, confidence intervals, point estimates etc.)
Results	10a	Participants - comprehensively describe<br>Flow of participants (recruitment, nonparticipation, cross-over and withdrawal, with reasons). Use figure to illustrate.<br>Population demographics (e.g. age, sex, relevant socioeconomic features, prognostic features etc.)<br>Any significant numerical differences should be highlighted	Results	9a	*Participants*<br>*Comprehensively describe*:<br>With reasons, the flow of participants (recruitment, nonparticipation, cross-over and withdrawal), using a figure to illustrate where appropriate<br>Population demographics (e.g. age, sex, relevant socioeconomic features, prognostic features etc.)<br>Any significant numerical differences across groups<br>If applicable, the longitudinal changes in participant flow/demographics over time
	10b	Participant comparison<br>Include table comparing baseline characteristics of cohort groups<br>Give differences, with statistical relevance<br>Describe any group matching, with methods		9b	Participant comparison<br>Include table comparing baseline characteristics of cohort groups, with statistical data included<br>Concisely, highlight the principal, significant findings<br>Describe any group matching, with methods
	10c	Intervention - comprehensively describe<br>Degree of novelty of intervention<br>Learning required for interventions<br>Any changes to interventions, with rationale and diagram, if appropriate			
	11a	Outcomes - comprehensively describe<br>Clinician-assessed and patient-reported outcomes for each group<br>Relevant photographs and imaging are desirable<br>Any confounding factors and state which ones are adjusted		9c	*Outcomes*<br>*Comprehensively describe*:<br>Clinician-assessed and patient-reported outcomes (e.g. questionnaires with quality-of-life scales) for each group<br>Expected versus attained outcomes, as assessed by the clinician*<br>Primary and secondary outcomes, as previously defined *(Item 8h)*<br>Details of when the outcomes were recorded (e.g. at how many months/years postoperatively)<br>Relevant photographs and imaging are desirable<br>Any confounding factors and state which ones are adjusted and how<br>Any changes to interventions, with rationale and diagram, if appropriate<br>**NB: reference relevant literature to inform expected outcomes*
	11b	Tolerance - comprehensively describe<br>Assessment of tolerability of exposure/intervention<br>Cross-over with explanation<br>Loss to follow-up (fraction and percentage), with reasons		9d	*Tolerance*<br>*Comprehensively describe*:<br>Assessment of tolerability of exposure/intervention within patient groups<br>Methods of measuring tolerance/adherence<br>If applicable, specific patient perspectives<br>Whether these results will have an impact on the long-term applicability of the findings in clinical practice<br>Loss to follow-up (fraction and percentage), with reasons
	11c	Complications - comprehensively describe<br>Adverse events and classify according to Clavien–Dindo classification*<br>Timing of adverse events<br>Mitigation for adverse events (e.g. blood transfusion, wound care, revision surgery etc.)<br>* Dindo D, Demartines N, Clavien P-A. Classification of Surgical Complications. A New Proposal with Evaluation in a Cohort of 6336 Patients and Results of a Survey. Ann Surg. 2002; 240(2): 205-213		9e	*Complications*<br>*Comprehensively describe*:<br>Adverse events, classified according to the Clavien–Dindo classification*<br>Timing of adverse events<br>Precautionary measures taken to prevent complications (e.g. antibiotic or venous thromboembolism prophylaxis)<br>Management of adverse events (e.g. blood transfusion, wound care, revision surgery etc.)<br>If applicable, whether the complication was reported to the national agency/pharmaceutical company<br>If applicable, specify whether any complications were discussed locally and the impact of such discussions (e.g. during team morbidity & mortality meetings)<br>State explicitly if there were no complications/adverse outcomes<br>**Dindo D, Demartines N, Clavien P-A. Classification of Surgical Complications. A New Proposal with Evaluation in a Cohort of 6336 Patients and Results of a Survey. Ann Surg. 2002; 240(2): 205-213*
	12	Key results - comprehensively describe<br>Key results with relevant raw data<br>Statistical analyses with significance<br>Include table showing research findings and statistical analyses with significance		9f	*Key results*<br>*Describe*:<br>Key findings, supported by relevant raw data and corresponding statistical analyses with significance
Discussion	13	Discussion - comprehensively describe<br>Conclusions and rationale<br>Reference to relevant literature<br>Implications for clinical practice	Discussion	10a	*Principal findings*<br>By referencing key, relevant literature throughout, comprehensively describe:<br>Summary of key findings and conclusions<br>Rationale behind conclusions drawn

Table 1

(*Continued*)

Comparison of STROCSS 2021 and proposed STROCSS 2024

**STROCSS 2021**			**Proposed STROCSS 2024**		
**Topic**	**Item**	**Guideline**	**Topic**	**Item**	**Guideline**
		Comparison to current gold standard of care<br>Relevant hypothesis generation			Comparison to current gold standard of care, current guidelines or similar research<br>Implications of findings for future clinical practice and guidelines<br>Relevant hypothesis generation
	14	Strengths and limitations - comprehensively describe<br>Strengths of the study<br>Weaknesses and limitations of the study and potential impact on results and their interpretation<br>Assessment and management of bias<br>Deviations from protocol, with reasons		10b	*Strengths and limitations*<br>Comprehensively describe:<br>Strengths of the study<br>Weaknesses and limitations of the study<br>Measures taken to overcome the limitations, if applicable<br>Potential impact on results and their interpretation<br>Assessment and management of bias<br>Deviations from protocol, with reasons stated
	15	Relevance and implications - comprehensively describe<br>Relevance of findings and potential implications for clinical practice<br>Need for and direction of future research		10c	*Relevance and implications*<br>Comprehensively describe:<br>Relevance of findings<br>Potential implications for future clinical practice and guidelines<br>Measures that can be taken to enhance the quality of research study<br>Need for and direction of future research
Conclusion	16	Conclusions<br>Summarise key conclusions<br>Outline key directions for future research		11	*Conclusions*<br>Summarise key conclusions, in a concise manner<br>Outline scope for and direction of future research
			Additional information	*12a-12d*	*Items 12a-12d of STROCSS 2024 correlate with items 4a-4d of STROCSS 2021.*
Declarations	17a	Conflicts of interest<br>Conflicts of interest, if any, are described	Declarations	13b	*Conflicts of interest*<br>Conflicts of interest, if any, are described
	17b	Funding<br>Sources of funding (e.g. grant details), if any, are clearly stated<br>Role of funder		13c	*Funding*<br>Sources of funding (e.g. grant details), if any, are clearly stated<br>Role of funder stated<br>Guarantor named
	17c	Contributorship<br>Acknowledge patient and public involvement in research; report the extent of involvement of each contributor		13a	*Contributorship*<br>Acknowledge any patient and/or public and/or professional involvement in research<br>Report the extent of involvement of each contributor, specifically stating what they contributed to (e.g. patient recruitment, defining research outcomes, dissemination of results etc.).
				13d	*Data sharing statement*<br>Explicitly state whether or not the datasets generated during study are available on request
