# Supplementary material for: Three Types of Collateral Arterial Supply to the Spleen After Spleen-Preserving Distal Pancreatectomies with Splenic Vessels Resection—How to Use This Knowledge for Organ(s) Preservation in Locally Advanced and Borderline Resectable Pancreatic Head Cancers Surgery—Hemodynamic, Surgical and Oncological Outcomes of 134 Spleen-Preserving Pancreatectomies
Source: Cancers (Basel). 2026 May 21;18(10):1675. doi: 10.3390/cancers18101675 (PMC13204045; doi:10.3390/cancers18101675)

Statistics to "Three types of collateral arterial supply to the spleen after spleen-preserving distal pancreatectomies with splenic vessels resection. How to use this knowledge for organ(s) preservation in locally advanced and borderline-resectable pancreatic head cancers surgery. Hemodynamic, Surgical and Oncological Outcomes of 134 spleen-preserving pancreatectomies."

Frequencies

Gender

		Frequency	Percent	Valid Percent	Cumulative Percent
Valid	f	23	56,1	56,1	56,1
	m	18	43,9	43,9	100,0
	Total	41	100,0	100,0	

Frequencies

Dindo-C

		Frequency	Percent	Valid Percent	Cumulative Percent
Valid	0-2	31	75,6	77,5	77,5
	≥3	9	22,0	22,5	100,0
	Total	40	97,6	100,0	
Missing	System	1	2,4		
Total		41	100,0		

Custom Tables

	Valid N	Mean	Standard Deviation	Median	Percentile 25	Percentile 75
BMI	41	23,45	4,29	23,00	20,00	25,00
Age	41	57,83	10,10	61,00	50,00	65,00

CA 19-9 перед началом лечения	35	308,69	352,15	236,00	89,00	435,00
CA 19-9 перед операцией	35	49,50	69,59	35,00	23,00	48,00
CA-19 Ratio	33	,68	2,00	,16	,09	,38
LN all	40	37,55	13,07	34,50	29,50	46,50
Tumor size, pathol, mm	40	34,62	13,30	35,50	26,00	41,00
LOS, days	41	16,39	4,72	16,00	13,00	19,00
OP time, min	40	560,50	146,64	565,00	470,00	665,00
Blood loss, ml	40	358,25	211,28	290,00	200,00	485,00
NACHT #	40	11,60	2,95	12,00	12,00	12,00
ACHT #	40	4,43	2,56	6,00	2,00	6,00

Frequencies

group

		Frequency	Percent	Valid Percent	Cumulative Percent
Valid	1	28	100,0	100,0	100,0

Custom Tables

	Valid N	Mean	Standard Deviation	Median	Percentile 25	Percentile 75
Time of follow-up	28	34,21	15,00	29,50	25,50	37,00

Kaplan-Meier

Case Processing Summary

Total N	N of Events	Censored	
		N	Percent

28	18	10	35,7%

Survival Table

	Time	Status	Cumulative Proportion Surviving at the Time		N of Cumulative Events	N of Remaining Cases
			Estimate	Std. Error		
1	18,000	yes	.	.	1	27
2	18,000	yes	,929	,049	2	26
3	19,000	yes	,893	,058	3	25
4	20,000	yes	,857	,066	4	24
5	22,000	yes	.	.	5	23
6	22,000	yes	,786	,078	6	22
7	23,000	yes	,750	,082	7	21
8	24,000	yes	,714	,085	8	20
9	24,000	no	.	.	8	19
10	25,000	yes	,677	,089	9	18
11	26,000	yes	,639	,091	10	17
12	27,000	no	.	.	10	16
13	28,000	yes	.	.	11	15
14	28,000	yes	,559	,096	12	14
15	28,000	no	.	.	12	13
16	30,000	yes	,516	,098	13	12
17	32,000	no	.	.	13	11
18	32,000	no	.	.	13	10
19	33,000	no	.	.	13	9
20	35,000	yes	.	.	14	8
21	35,000	yes	,401	,104	15	7
22	35,000	no	.	.	15	6
23	40,000	yes	,335	,106	16	5
24	40,000	no	.	.	16	4
25	44,000	yes	,251	,108	17	3
26	45,000	yes	,167	,099	18	2
27	56,000	no	.	.	18	1
28	93,000	no	.	.	18	0

Means and Medians for Survival Time

Mean			Median		
Estimate	Std. Error	95% Confidence Interval	Estimate	Std. Error	95% Confidence Interval

		Lower Bound	Upper Bound			Lower Bound	Upper Bound
41,066	5,911	29,480	52,653	35,000	4,631	25,922	44,078

Percentiles

25,0%		50,0%		75,0%	
Estimate	Std. Error	Estimate	Std. Error	Estimate	Std. Error
45,000	2,962	35,000	4,631	23,000	2,267

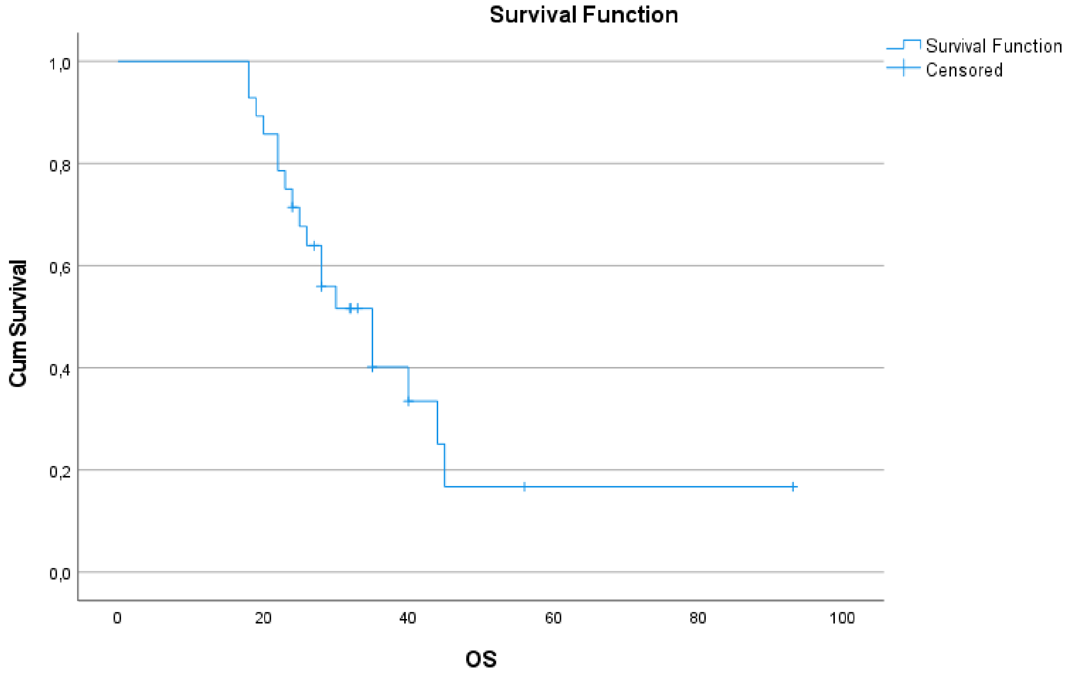

Kaplan-Meier

Case Processing Summary

Total N	N of Events	Censored	
		N	Percent
28	24	4	14,3%

Survival Table

	Time	Status	Cumulative Proportion Surviving at the Time		N of Cumulative Events	N of Remaining Cases
			Estimate	Std. Error		
1	11,000	yes	,964	,035	1	27
2	12,000	yes	.	.	2	26
3	12,000	yes	.	.	3	25
4	12,000	yes	,857	,066	4	24
5	14,000	yes	.	.	5	23
6	14,000	yes	,786	,078	6	22
7	16,000	yes	,750	,082	7	21
8	17,000	yes	,714	,085	8	20
9	18,000	yes	.	.	9	19
10	18,000	yes	,643	,091	10	18
11	19,000	yes	,607	,092	11	17
12	20,000	yes	,571	,094	12	16
13	21,000	yes	.	.	13	15
14	21,000	yes	,500	,094	14	14
15	22,000	yes	.	.	15	13
16	22,000	yes	,429	,094	16	12
17	25,000	yes	,393	,092	17	11
18	26,000	yes	.	.	18	10
19	26,000	yes	.	.	19	9
20	26,000	yes	,286	,085	20	8
21	28,000	yes	.	.	21	7
22	28,000	yes	,214	,078	22	6
23	29,000	yes	,179	,072	23	5
24	32,000	no	.	.	23	4
25	35,000	yes	,134	,067	24	3
26	35,000	no	.	.	24	2
27	56,000	no	.	.	24	1
28	93,000	no	.	.	24	0

Means and Medians for Survival Time

Mean				Median			
		95% Confidence Interval				95% Confidence Interval	
Estimate	Std. Error	Lower Bound	Upper Bound	Estimate	Std. Error	Lower Bound	Upper Bound
30,339	4,896	20,744	39,935	21,000	1,323	18,407	23,593

Percentiles

25,0%		50,0%		75,0%	
Estimate	Std. Error	Estimate	Std. Error	Estimate	Std. Error
28,000	1,447	21,000	1,323	16,000	2,291

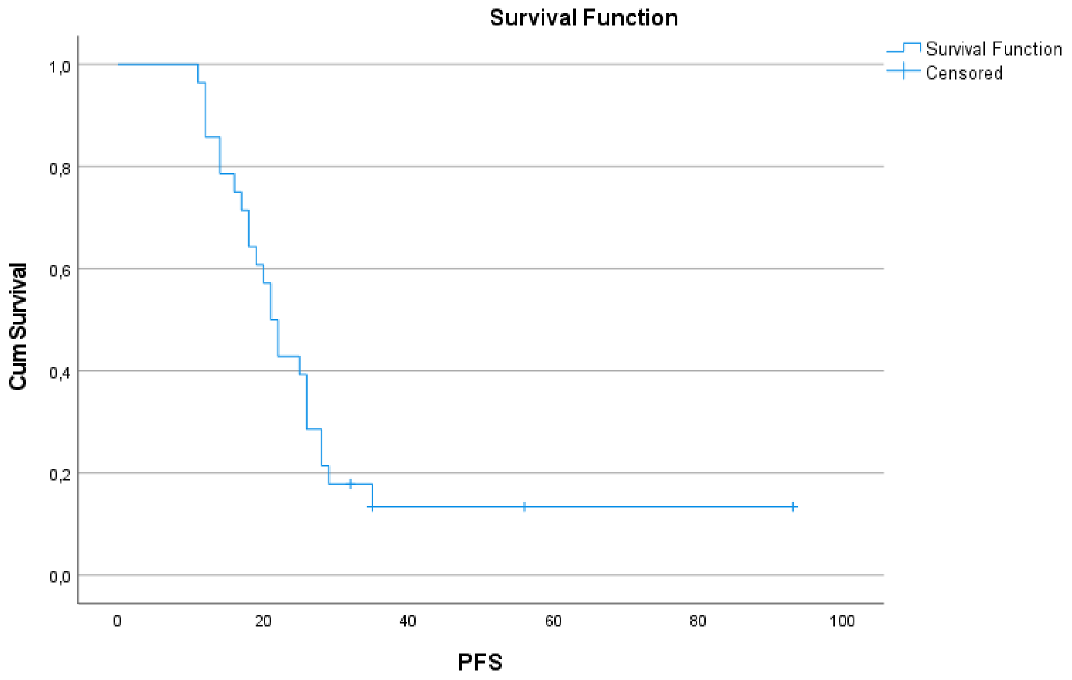

Supplement: Supplementary file 1 [file cancers-18-01675-s001.zip › File S5. Statistics. TP and PD for LA PHDAC Egorov et al.pdf]
